# Relationship between gut microbiota and rheumatoid arthritis: A bibliometric analysis

**DOI:** 10.3389/fimmu.2023.1131933

**Published:** 2023-03-01

**Authors:** Ying Dong, Jianling Yao, Qingyue Deng, Xianxian Li, Yingyu He, Xueyang Ren, Yuan Zheng, Ruolan Song, Xiangjian Zhong, Jiamu Ma, Dongjie Shan, Fang Lv, Xiuhuan Wang, Ruijuan Yuan, Gaimei She

**Affiliations:** ^1^ School of Chinese Materia Medica, Beijing University of Chinese Medicine, Beijing, China; ^2^ Peking University HuiLongGuan Clinical Medical School, Beijing Huilongguan Hospital, Beijing, China

**Keywords:** bibliometrics, rheumatoid arthritis, gut microbiota, Bibliometrix, VOSviewer

## Abstract

**Introduction:**

Rheumatoid arthritis (RA) is a multifactorial autoimmune disease. Recently, growing evidence demonstrates that gut microbiota (GM) plays an important role in RA. But so far, no bibliometric studies pertaining to GM in RA have ever been published. This study attempts to depict the knowledge framework in this field from a holistic and systematic perspective based on the bibliometric analysis.

**Methods:**

Literature related to the involvement of GM in RA was searched and picked from the Web of Science Core Collection (WOSCC) database. The annual output, cooperation, hotspots, research status and development trend of this field were analyzed by bibliometric software (VOSviewer and Bibliometricx).

**Results:**

255 original research articles and 204 reviews were included in the analysis. The articles in this field that can be retrieved in WOSCC were first published in 2004 and increased year by year since then. 2013 is a growth explosion point. China and the United States are the countries with the most contributions, and Harvard University is the affiliation with the most output. Frontiers in Immunology (total citations = 603) is the journal with the most publications and the fastest growth rate. eLife is the journal with the most citations (total citations = 1248). Scher, Jose U. and Taneja, Veena are the most productive and cited authors. The research in this field is mainly distributed in the evidence, mechanism and practical application of GM participating in RA through the analysis of keywords and documents. There is sufficient evidence to prove the close relationship between GM and RA, which lays the foundation for this field. This extended two colorful and tender branches of mechanism research and application exploration, which have made some achievements but still have broad exploration space. Recently, the keywords ”metabolites“, ”metabolomics“, ”acid“, ”b cells“, ”balance“, ”treg cells“, ”probiotic supplementation“ appeared most frequently, which tells us that research on the mechanism of GM participating in RA and exploration of its application are the hotspots in recent years.

**Discussion:**

Taken together, these results provide a data-based and objective introduction to the GM participating in RA, giving readers a valuable reference to help guide future research.

## 1 Introduction

Rheumatoid arthritis (RA) is a chronic autoimmune disease with a high disability rate. It is characterized by destructive and symmetrical joint diseases and synovitis, which seriously threaten human health (1). The pathogenesis of RA is very complex, with both genetic and environmental factors involved (2). More than 100 trillion microbes are inhabiting human bodies, the majority of which reside in the gut (3). With the progress of bacterial DNA sequencing technologies, the relationship between intestinal bacteria and RA is gradually revealed, suggesting that gut microbiota (GM) plays a notable role in RA (2, 4). It is reported that there is a significant difference in GM between RA patients and healthy individuals, and the alteration of GM can affect the manifestation of RA (5, 6).

In recent years, studies on the relationship between RA and GM have been carried out in large quantities. More and more researchers devote themselves to this field. The systematic and holistic literature review will help better understand the current research situation and select research directions. Bibliometrics is an efficient method for analyzing the development trend, research progress, hotspots, discipline knowledge structure and its dynamic evolution relationship in a field by quantitatively analyzing literature materials through mathematical and statistical methods (7, 8). So far, bibliometric analyses have not been seen in the field of GM in RA. In this study, we used bibliometric analysis to sort out and analyze the annual output, cooperation, hotspots, research structure and development trend of this field from a holistic and systematic perspective. We hope that this study can provide effective information to understand the development status and trends in the field of GM in RA and help to better carry out future work.

## 2 Materials and method

### 2.1 Data collection and filtration

We conducted a comprehensive literature search in the Web of Science Core Collection (WOSCC) (https://www.webofscience.com/wos/woscc/basic-search). Considering the rapid update of the database, the literature retrieval was carried out in a single day (November 14, 2022). The publication period in this study was set from 1985 to 2022. The search terms were presented as follows: Topic = “rheumatoid arthritis” AND Topic = “gut microbiota” or “intestinal microbiota” or “fecal microbiota” or “gastrointestinal microbiota” or “gut microbiome” or “intestinal microbiome” or “fecal microbiome” or “gastrointestinal microbiome” or “intestinal bacteria” or “gut bacteria” or “fecal bacteria” or “gastrointestinal bacteria” or “intestinal flora” or “gut flora” or “fecal flora” or “gastrointestinal flora” or “gut microflora” or “intestinal microflora” or “fecal microflora” or “gastrointestinal microflora”. Document types for articles and reviews in English were selected. In the articles initially searched, the reported contents of some articles were not related to RA in GM, which were manually excluded. Finally,459 results were obtained. The list of documents collected is provided in the “Collected documents list” sheet in the **Supplementary Material 1**.

### 2.2 Bibliometric analysis

The data on annual publications output came from the section “Analyze Results” in WOSCC. VOSviewer v1.6.18.0 (9) was used to perform cooperation relationships between countries/regions or affiliations, and keywords co-occurrence network. Bibliometricx (10) was used to analyze the publication and cooperation of countries/regions, the author’s output timeline, the citation of the articles, topic migration and the thematic map. All raw data for the bibliometric analysis can be obtained from https://doi.org/10.6084/m9.figshare.c.6381747.v1.

## 3 Results

### 3.1 Quantities and trend of publications output

**Figure 1:**
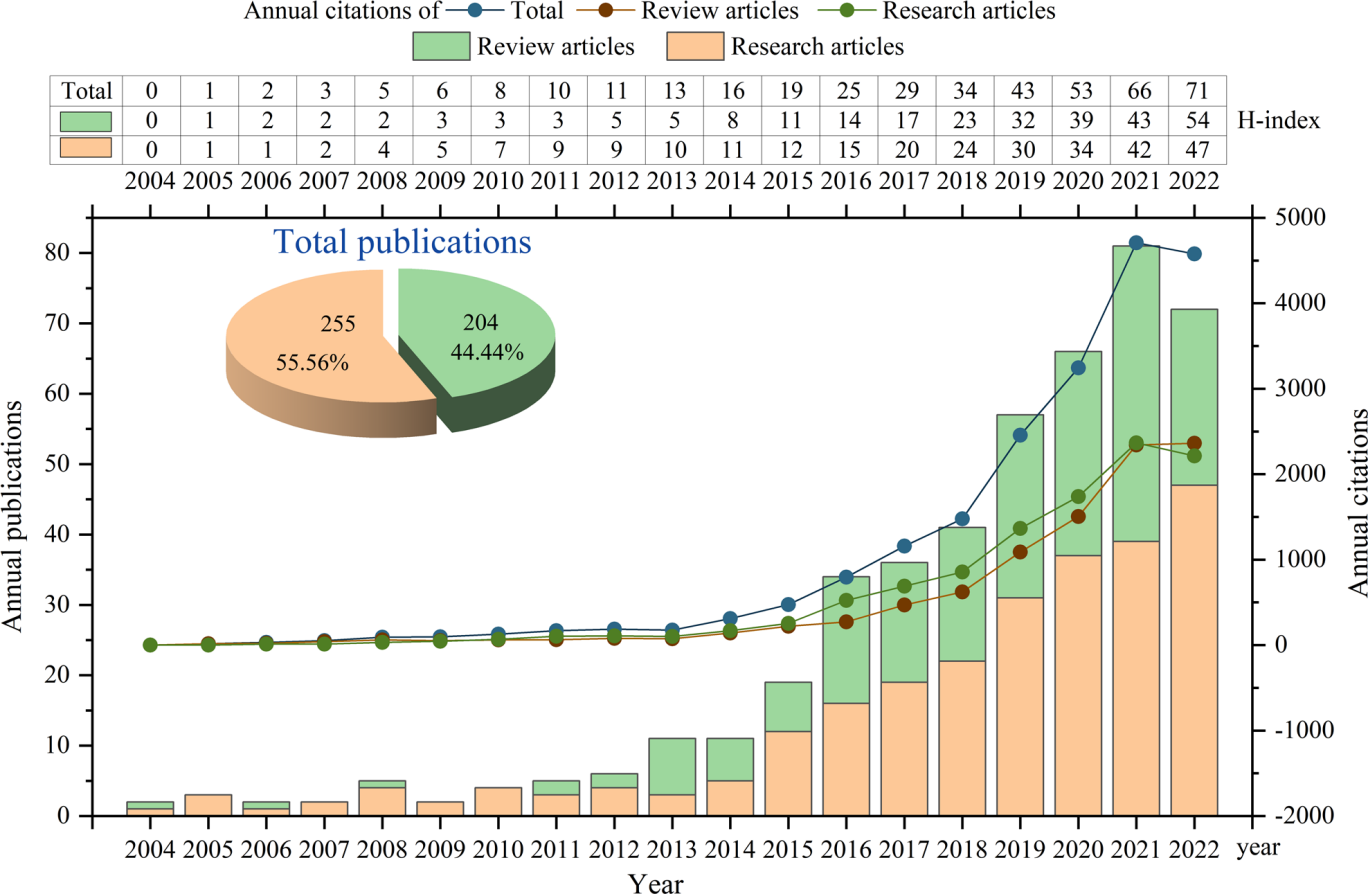
Quantities and trend of publications output and annual citations of GM in RA from 2004 to 2022.

In this study, a total of 459 pieces of literature related to the involvement of GM in RA were included in the analysis. Among them, there were 255 original research articles and 204 reviews (**Figure 1**). Subsequently, these articles were used to access the discoveries and trends of the relationship between RA and GM. The total citations of these articles were 20153, including 10659 for research articles and 9494 for reviews. The annual output, citations and H-index in this field were shown in **Figure 1**. As can be seen, the earliest original research article was published in 2005, while the first report was a review article in 2004. The total number of citations and publications per year consistently did not change much from 2004 to 2012, and then reached a turning point, showing continuous growth from 2013. The H-index increased year by year, reaching 71 in 2022.

### 3.2 Analysis of countries/regions

**Figure 2:**
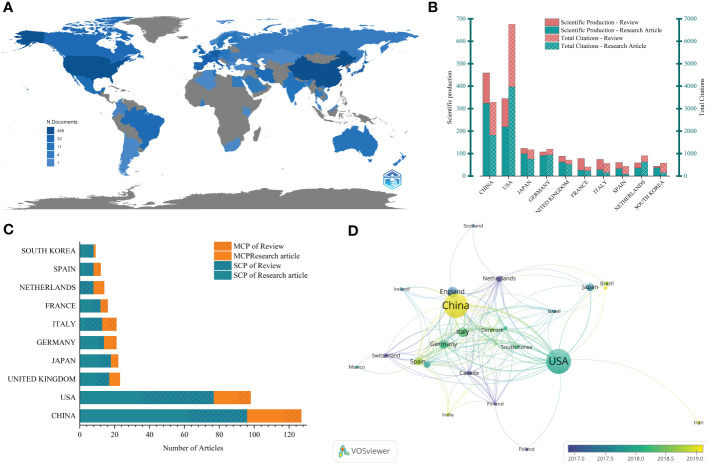
Visualization of countries/regions analysis. **(A)** Geographical distribution of global outputs; **(B)** The top 10 countries/regions in the output list and their citations (all authors included); **(C)** The top 10 countries/regions in output (corresponding authors included), MCP, Multiple Country Publications; SCP, Single Country Publications; **(D)** Cooperation networks across countries.

These publications came from 64 countries/regions. The Bibliometrix package was used to measure the output of countries/regions by counting the number of “author’s appearances by country affiliations”. There were two modes, one was that all authors were included in the scope (**Figure 2A, B**), and the other was that only corresponding authors were considered (**Figure 2C**). The top 10 countries/regions according to the two models are shown in **Figures 2B, C** respectively. The results showed that in all 459 articles, the number of articles and citations in China and the United States was significantly higher than that in other countries/regions. The output of other countries was less than 50 articles, and 38 countries/regions had less than 5 articles.

The cooperation between countries was shown in **Figures 2C, D**. In **Figure 2C**, Multiple Country Publications (MCP) referred to the number of articles co-signed with authors from other countries/regions, while Single Country Publications (SCP) referred to the number of articles whose authors were all from the same country/region. The proportion of MCP can reflect the current situation of international cooperation and the exchange of academic research in this field. It could be seen that academic research in this field in most countries/regions was mainly carried out locally. However, it also had a certain foundation for international cooperation, with MCP accounting for about one-third. The cooperation network between countries/regions was drawn through Vosviewer (**Figure 2D**), which told us that international cooperation was mainly carried out by countries/regions actively studying this field. In the cooperation network, China and the United States had the closest cooperation and showed their core position. The two have carried out extensive cooperation with Germany, the UK, Italy, Japan and other countries/regions.

### 3.3 Analysis of affiliations

**Figure 3:**
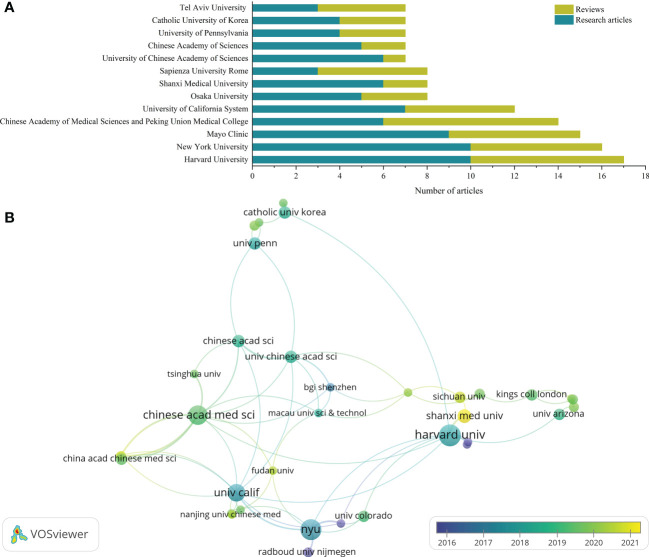
Visualization of affiliations analysis. **(A)** The top 10 affiliations with the most publications; **(B)** Cooperation networks across affiliations.

Publications in the study of GM in RA came from 845 affiliations, most of which were universities. The top 10 affiliations in the number of academic output in this field were shown in **Figure 3A**. Harvard University had the highest number of publications (10 research articles and 7 reviews), followed by New York University (10 research articles and 6 reviews) and Mayo Clinic (9 research articles and 6 reviews). About two-thirds of the affiliations only had one article.

As shown in the affiliation cooperation network (**Figure 3B**), the cooperation between affiliations was mainly centered on Harvard University, New York University, University of California System and Chinese Academy of Medical Sciences. Although Mayo Clinic ranks among the top three in academic output in this field, its cooperation with other affiliations was not frequent, so it was not included in the network.

### 3.4 Analysis of authors

A total of 2472 authors participated in the study of GM in RA. It is worth noting that the analysis results of research articles and reviews differed greatly. **Table 1** listed the authors who have published 4 or more research articles with a total citation of more than 100, as well as the four authors with fewer publications but significant total citations. **Table 2** listed the authors who have published more than 2 reviews or whose total citations of more than 200. From the results of research and review articles, it could be seen that Scher, Jose U. from New York University in the USA and Taneja, Veena from Mayo Clinic in the USA had the most prolific output and citations. The number of publications by most authors had not a wide gap.

**Table 1:** The top authors with the most research articles and total citations.

NO.	Author	Country/region	Affiliation	Research articles	Reviews	Total citations of research articles
1	Taneja, Veena	USA	Mayo Clinic	8	5	713
2	Scher, Jose U.	USA	New York University	6	7	1299
3	Abdollahi-Roodsaz, Shahla	Netherlands	Radboud University Medical Center	5	1	511
4	Koenders, Marije I.	Netherlands	Radboud University Medical Center	5	2	511
5	Strowig, Till	Germany	Helmholtz Association	5	0	572
6	van den Berg, Wim B.	Netherlands	Radboud University Medical Center	4	0	504
7	Maeda, Yuichi	Japan	Osaka University	4	2	433
8	Takeda, Kiyoshi	Japan	Osaka University	4	2	433
9	Zaiss, Mario M.	Germany	Universitäts Klinikum Erlangen	4	3	429
10	Davis, John M.	USA	Mayo Clinic	4	0	405
11	Wu, Hsin-Jung Joyce	USA	University of Arizona	4	2	251
12	Manasson, Julia	USA	New York University	4	1	174
13	Rogier, Rebecca	Netherlands	Radboud University Medical Center	4	1	144
14	van der Kraan, Peter M.	Netherlands	Radboud University Medical Center	4	0	144
15*	Abramson, Steven B.	USA	New York University	3	4	1191
16*	Ubeda, Carles	Spain	University of Valencia	3	1	1163
17*	Huttenhower, Curtis	USA	Harvard University	2	1	1587
18*	Littman, Dan R.	USA	New York University	2	0	1417

*authors with less publications but significant total citations.

**Table 2:** The top authors with the most reviews and total citations.

NO.	Author	Country/region	Affiliation	Reviews	Research articles	Total citations of reviews
1	Scher, Jose U.	USA	New York University	7	6	774
2	Taneja, Veena	USA	Mayo Clinic	5	8	252
3	Abramson, Steven B.	USA	New York University	4	3	474
4	Shoenfeld, Yehuda	Israel	Tel Aviv University	4	1	266
5	Fonseca, Joao Eurico	Portugal	Universidade de Lisboa	4	0	85
6	Zhang, Xuan	China	Chinese Academy of Medical Sciences and Peking Union Medical College	3	1	209
7	Guerreiro, Catarina Sousa	Portugal	Universidade de Lisboa	3	0	80
8	Kuhn, Kristine A.	USA	University of Colorado - Anschutz Medical Campus	3	3	76
9	Niu, Haitao	China	Chinese Academy of Medical Sciences and Peking Union Medical College	2	0	253
10	Shi, Na	China	Chinese Academy of Medical Sciences and Peking Union Medical College	2	0	253
11	van Eden, Willem	Netherlands	Utrecht University	2	0	217

**Figure 4:**
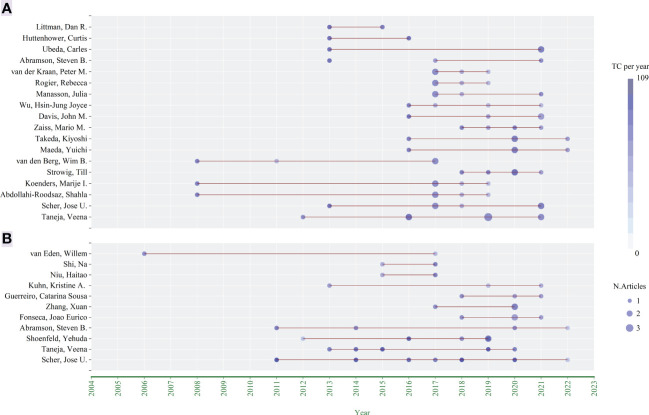
Authors’ production in over time. **(A)** Research articles; **(B)** Reviews.

According to the year when each author published the articles, the number of articles and the total citations per year of the article published in that year, the chart “Authors’ Production Over Time” (**Figures 4A, B**) was drawn by Bibliometrix. Taneja, Veena and Scher, Jose U. have been studying in this field for more than 10 years and were continuing (there were still their new publications last year). There were two pivotal periods, 2011-2013 and 2016-2017, which were the concentrated periods for these authors to publish their first articles.

### 3.5 Analysis of journals

**Figure 5:**
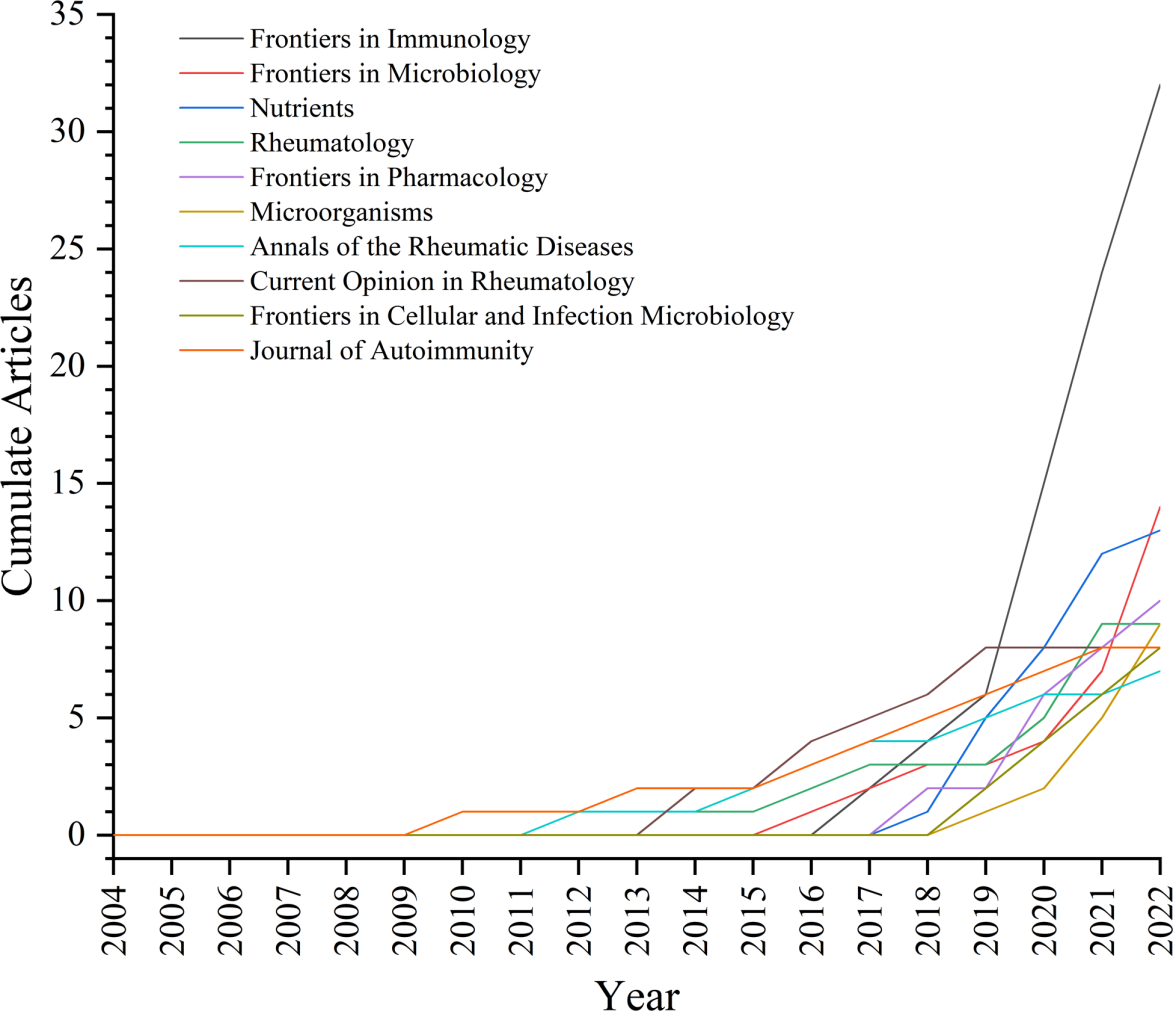
Journals’ production in over time.

A total of 210 journals have published manuscripts in this field. The top 10 journals in the total number of relevant publications were listed in **Table 3**. About 26.36% of the articles were published in these journals. *Frontiers in Immunology* (32 publications, impact factor (IF) = 8.786) published the most papers in this field, followed by *Frontiers in Microbiology* (14 publications, IF = 6.064) and *Nutrients* (13 publications, IF = 6.706). Seven of the top 10 journals began to focus on this field after 2013 (**Figure 5**). **Table 4** listed the top 10 journals with the most citations, which was quite different from the list of published quantity (**Table 3**). *eLife* (1248 citations, IF = 8.713), *Nature Medicine* (847 citations, IF = 87.241), and *Cell* (824 citations, IF = 66.85) were the most cited journals.

**Table 3:** The top 10 journals with the most articles.

Journal	Total Articles	Research Articles	Reviews	Total Citations	IF (2022)	Start Year
Frontiers in Immunology	32	14	18	603	8.786	2017
Frontiers in Microbiology	14	8	6	343	6.064	2016
Nutrients	13	2	11	306	6.706	2018
Rheumatology	11	8	3	163	7.046	2010
Frontiers in Pharmacology	10	6	4	127	5.988	2018
Microorganisms	9	5	4	61	4.926	2019
Annals of the Rheumatic Diseases	8	8	0	483	27.973	2010
Current Opinion in Rheumatology	8	0	8	254	4.941	2014
Frontiers in Cellular and Infection Microbiology	8	6	2	130	6.073	2019
Journal of Autoimmunity	8	4	4	566	14.511	2010

**Table 4:** The top 10 journals with the most citations.

Journal	Total Articles	Research Articles	Reviews	Total Citations	IF (2022)	Start Year
eLife	2	2	0	1248	8.713	2013
Nature Medicine	1	1	0	847	87.241	2015
Cell	2	2	0	824	66.85	2015
Journal of Clinical Investigation	3	2	1	630	19.456	2008
Arthritis & Rheumatogy	6	6	0	628	15.483	2016
Immunology	3	0	3	621	7.215	2017
Frontiers in Immunology	32	14	18	603	8.786	2017
Journal of Autoimmunity	8	4	4	566	14.511	2010
Immunology Letters	3	1	2	505	4.23	2004
Annals of the Rheumatic Diseases	8	8	0	483	27.973	2010

### 3.6 High-cited articles

Because of the distinct types of original research and review articles and the different concerns of researchers on the two, we conducted citation analysis separately. There were two concepts, “Global Citations (GCS)” and “Local Citations (LCS)” in Bibliometrix. GCS meant the total citations of this article in WOSCC, while LCS meant citations in our collections. In this study, LCS reflected the influence of an article in the field of GM in RA. The top 10 highly local-cited research and review articles were presented in **Tables 5**, **6**. In original research articles, “Zhang X, 2015, Nat Med” and “Scher JU, 2013, eLife” had not only a large number of GCS but also a lot of LCS in this field, indicating that the two had a great influence in this field. In reviews, “Scher JU, 2011, Nat Rev Rheumatol”, “Brusca SB, 2014, Curr Opin Rheumatol” and “Horta-Baas G, 2017, J Immunol Res” had the top 3 LCS.

**Table 5:** The top 10 highly local cited research articles.

NO.	Document	Title	DOI	Year	LCS	GCS
1	Zhang X, 2015, Nat Med	The oral and gut microbiomes are perturbed in rheumatoid arthritis and partly normalized after treatment	10.1038/nm.3914	2015	107	847
2	Scher JU, 2013, eLife	Expansion of intestinal Prevotella copri correlates with enhanced susceptibility to arthritis	10.7554/eLife.01202	2013	102	1090
3	Chen J, 2016, Genome Med	An expansion of rare lineage intestinal microbes characterizes rheumatoid arthritis	10.1186/s13073-016-0299-7	2016	62	361
4	Vaahtovuo J, 2008, J Rheumatol	Fecal microbiota in early rheumatoid arthritis	https://www.jrheum.org/content/35/8/1500	2008	57	301
5	Maeda Y, 2016, Arthritis Rheumatol	Dysbiosis Contributes to Arthritis Development *via* Activation of Autoreactive T Cells in the Intestine	10.1002/art.39783	2016	45	313
6	Liu XF, 2013, Current Microbiology	Analysis of Fecal Lactobacillus Community Structure in Patients with Early Rheumatoid Arthritis	10.1007/s00284-013-0338-1	2013	35	134
7	Liu XF, 2016, Scientific Reports	Role of the Gut Microbiome in Modulating Arthritis Progression in Mice	10.1038/srep30594	2016	31	102
8	Abdollahi-Roodsaz S, 2008, The Journal of Clinical Investigation	Stimulation of TLR2 and TLR4 differentially skews the balance of T cells in a mouse model of arthritis	10.1172/JCI32639	2008	30	367
9	Picchianti-Diamanti A, 2018, Int J Mol Scis	Analysis of Gut Microbiota in Rheumatoid Arthritis Patients: Disease-Related Dysbiosis and Modifications Induced by Etanercept	10.3390/ijms19102938	2018	29	92
10	Vaghef-Mehrabany E, 2014, Nutrition	Probiotic supplementation improves inflammatory status in patients with rheumatoid arthritis	10.1016/j.nut.2013.09.007	2014	24	156

**Table 6:** The top 10 highly local cited review articles.

NO.	Document	Title	DOI	Year	LCS	GCS
1	Scher JU, 2011, Nat Rev Rheumatol	The microbiome and rheumatoid arthritis	10.1038/nrrheum.2011.121	2011	27	296
2	Brusca SB, 2014, Curr Opin Rheumatol	Microbiome and mucosal inflammation as extra-articular triggers for rheumatoid arthritis and autoimmunity	10.1097/BOR.0000000000000008	2014	18	133
3	Horta-Baas G, 2017, J Immunol Res	Intestinal Dysbiosis and Rheumatoid Arthritis: A Link between Gut Microbiota and the Pathogenesis of Rheumatoid Arthritis	10.1155/2017/4835189	2017	15	136
4	De Oliveira GLV, 2017, Immunology	Intestinal dysbiosis and probiotic applications in autoimmune diseases	10.1111/imm.12765	2017	13	150
5	Maeda Y, 2017, J Clin Med	Role of Gut Microbiota in Rheumatoid Arthritis	10.3390/jcm6060060	2017	11	118
6	Zhong DL, 2018, Clinical Rheumatology	The role of gut microbiota in the pathogenesis of rheumatic diseases	10.1007/s10067-017-3821-4	2018	11	45
7	Bodkhe R, 2019, Therapeutic Advances in Musculoskeletal Disease	The role of microbiome in rheumatoid arthritis treatment	10.1177/1759720X19844632	2019	11	56
8	Guerreiro CS, 2018, Frontiers in Medicine	Diet, Microbiota, and Gut Permeability—The Unknown Triad in Rheumatoid Arthritis	10.3389/fmed.2018.00349	2018	10	56
9	Maeda Y, 2019, Exp Mol Med	Host–microbiota interactions in rheumatoid arthritis	10.1038/s12276-019-0283-6	2019	10	59
10	Catrina AI, 2016, Rheumatology	Gene, environment, microbiome and mucosal immune tolerance in rheumatoid arthritis	10.1093/rheumatology/keu469	2016	9	63

### 3.7 Keywords co-occurrence network

**Figure 6:**
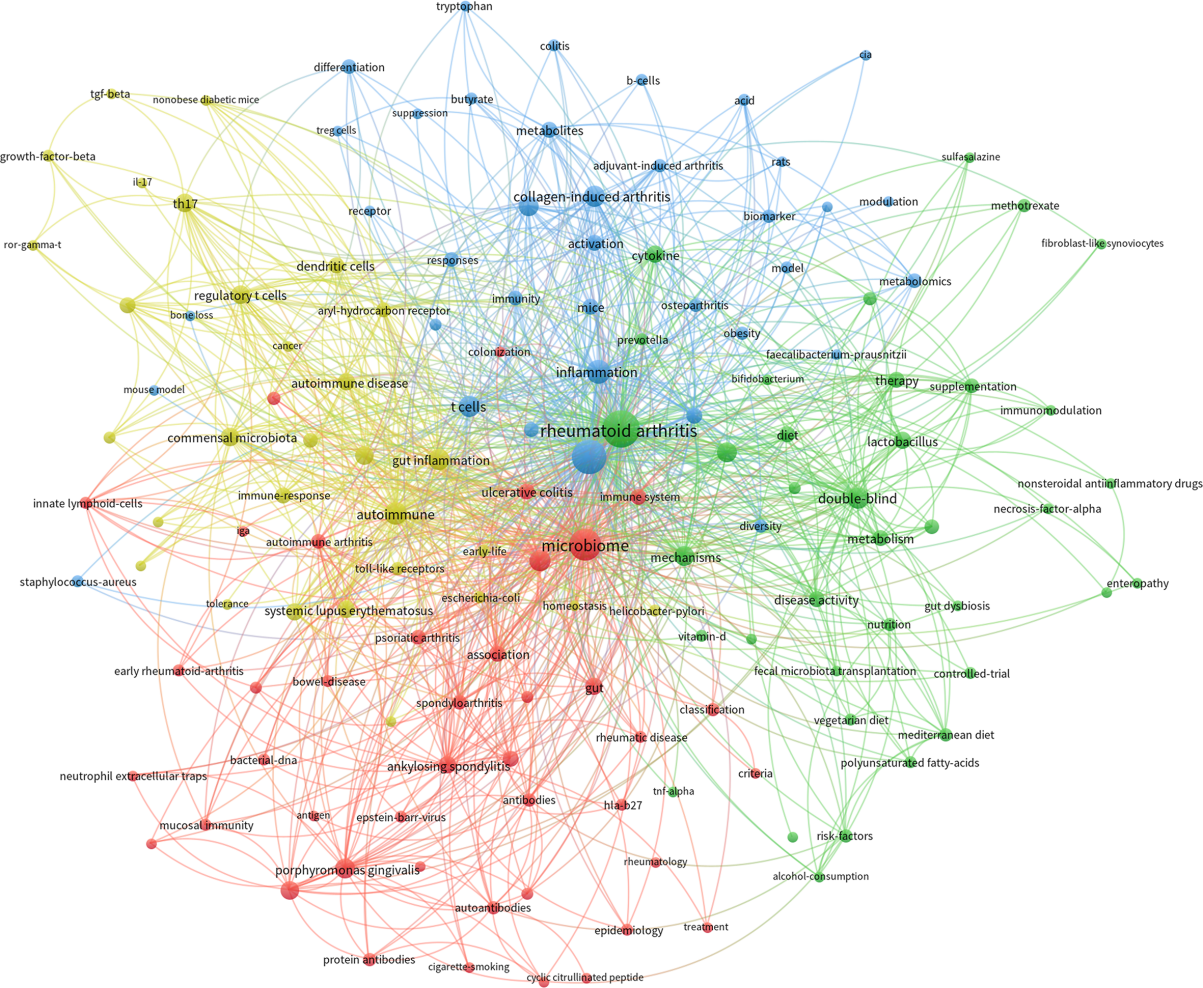
Visualization map of keywords co-occurrence network according to clusters.

Keywords co-occurrence network of papers of the GM in RA was conducted by Vosviewer software. Considering the readability and aesthetics of the graph, the minimum occurrence of keywords in these articles was set to 5, and 146 keywords were included in the co-occurrence network. The keywords of these articles were divided into four clusters with different colors (**Figure 6**). The four clusters of the keywords co-occurrence network had the characteristics of overlap and intersection, which indicated that the research in this field was not scattered and isolated.

The red cluster included 45 nodes, in which the keywords were “dysbiosis”, “association”, “immune system”, “classification”, “bacterial-dna”, “early rheumatoid-arthritis”, “mucosal immunity”, etc. This cluster was the commonly concerned theme in this field, mainly focusing on exploring the association between GM and RA, especially the alteration of GM in different RA disease conditions.

The green cluster included 37 nodes, reflecting the researchers’ exploration of regulating GM and thus affecting the condition of RA. It could be seen that there were mainly three paths being tried. The first was probiotic supplementation, suggested by keywords “probiotic”, “lactobacillus”, “probiotic supplementation”, “prevotella”, “fecal microbiota transplantation”, “bifidobacterium” and so on. Then there was dietary intervention, which could be extracted from keywords “diet”, “mediterranean diet”, “nutrition”, “vegetarian diet”, etc. The last was the redevelopment of RA drugs in terms of GM, as shown by keywords such as “methotrexate”, “nonsteroidal antiinflammatory drugs”, “proton pump inhibitors” and “sulfasalazine”.

The blue cluster included 37 nodes, mainly covering the keywords in the experimental research of GM in RA. It could be seen that in related experiments, the commonly used experimental animals were “mice” and “rats”, and the universally applied model was “collagen-induced arthritis” (CIA). “Chain fatty-acids”, “metabolites”, “metabolomics”, “butyrate”, “biomarker”, “t cells” and “b cells” were high-frequency keywords in this cluster, which indicated that metabolome was a commonly used method and the role of short chain fatty acids in the process of GM in RA were widely concerned.

The yellow cluster included 30 nodes. This cluster contained keywords such as “gut inflammation”, “regulatory t cells”, “commensal microbiota”, “th17”, “dendritic cells”, “immune-response”, “toll-like receptors”, “genome-wide association”, “growth-factor-beta”, and “il-17”. It could be seen that this cluster mainly involved the immune mechanism of intestinal bacteria participating in rheumatoid arthritis.

### 3.8 Hotspots and topic migration

**Figure 7:**
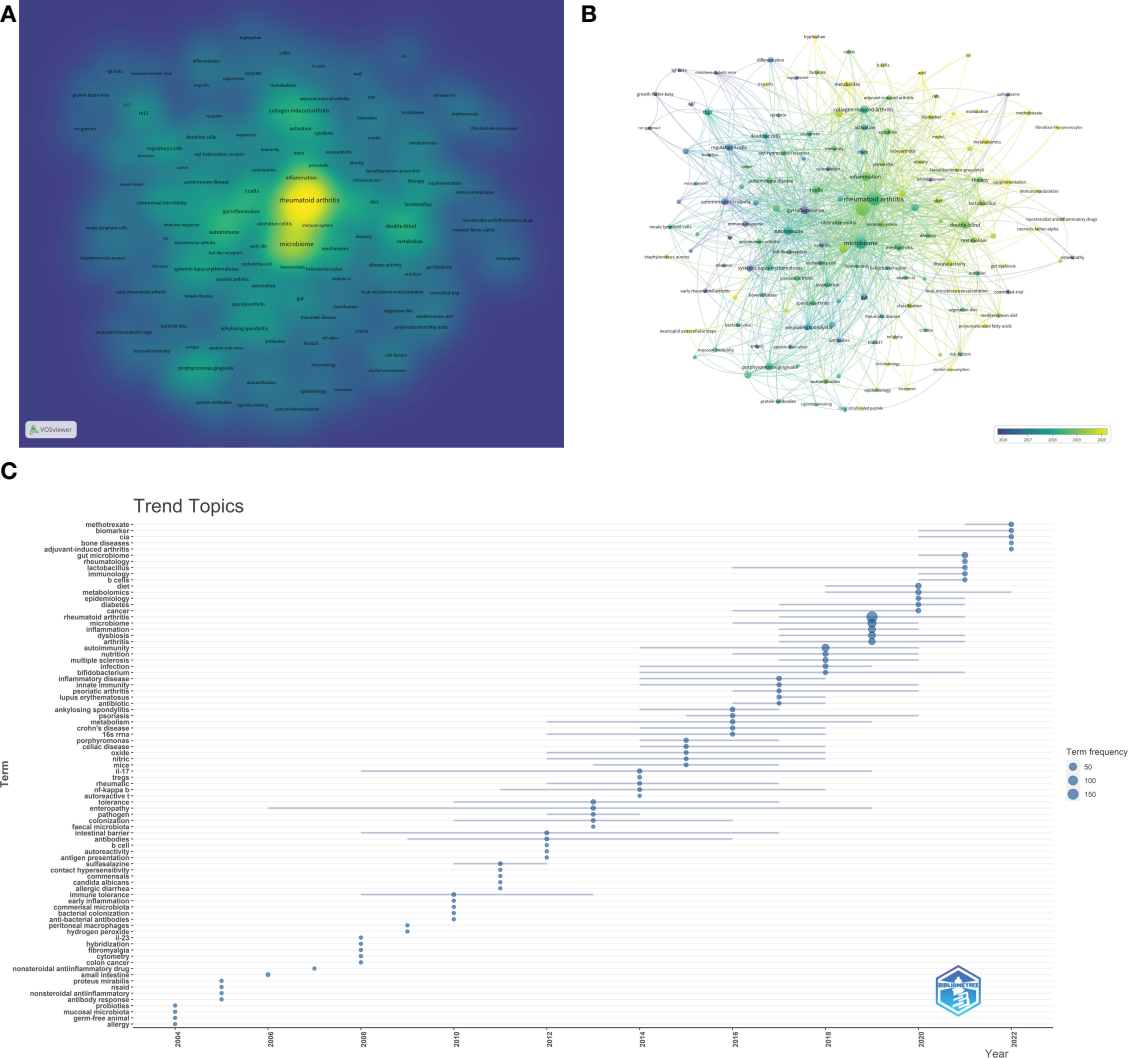
Analysis results of hotspot and topic migration in the field of GM in RA. **(A)** The density visualization of keywords co-occurrence network. **(B)** The timestamp visualization of keywords co-occurrence network. **(C)** The historical migration of research hotspots.

The density visualization of the keywords co-occurrence network was shown in **Figure 7A**. We can see that the research contents of these articles were around “rheumatoid arthritis” and “gut microbiome”. The hot keywords included “microbiome”, “autoimmune”, “inflammation”, “gut inflammation”, “dysbiosis”, “collagen-induced arthritis”, “double-blind”, “t cells”, “porphyromonas gingivalis”, “mechanisms”, “regulatory t cells”, “probiotic”, “chain fatty-acids”, “th17” and so on. Some pivotal information could be revealed from these hot keywords. For example, RA is an autoimmune disease and most of the studies of GM in RA were carried out from the perspective of immune and inflammatory response. And cell-mediated immunity mediated by T cells was a research hotspot. At present, no animal model can perfectly simulate human rheumatoid arthritis, but it can be seen from **Figure 6A** that collagen-induced arthritis (CIA) model was the most commonly used animal model, indicating that this model has been recognized by most researchers in this field. In mechanism research, the chain fatty-acids produced by GM and the influence of GM on Th17 cells were the focus of attention.

In **Figure 7B**, each node was marked with different colors according to the average time multiple of the keyword. We can see that the average occurrence time of most high-frequency keywords was after 2015, which indicated that this was a burgeoning field. Some keywords such as “metabolites”, “metabolomics”, ‘acid’, “b cells”, “balance”, “treg cells”, “probiotic supplementation”, etc., have appeared in about 2020 on average. This suggested that the research hotspots in recent years was mainly to study the mechanism of GM participating in RA and explore its application (such as probiotic supplementation).

To better take in the historical changes of research hotspots in this field, the “Trend Topics” analysis was carried out by the Bibliometric package. As shown in **Figure 7C**, in the early stage, the output was very low. Until 2013, the continuity of high-frequency keywords was not good every year. In 2013 and 2014, the high-frequency keywords were “fecal microbiota”, “nf-kappa b”, “tregs”, “il-17 (interleukin-17)”, “autoreactive t”, “oxide”, etc., suggesting that a large number of studies focused on the function of immune cells to explore the involvement of GM in the process of RA. High-frequency keywords in the documents from 2015 to 2017 included autoimmune diseases such as “psoriatic arthritis”, “lupus erythematosus”, “ankulosing spondylitis” and “psoriasislupus erythematosus”, and immune bowel diseases such as “crohn’s disease” and “celiac disease”. Similar to RA, these diseases are closely related to immune function and GM, and some pathogenesis and mechanisms have common points, which could provide novel ideas for the research of GM participating in RA. Since 2018, high-frequency keywords such as “metabolomics”, “biomarker”, “immunology”, “b cells”, “cia”, “adjuvant-induced arthritis”, “diet” and “lactobacillus” have emerged, indicating that the research hotspots in recent years were the mechanism and application of GM participating in RA, which was consistent with the result in **Figure 7B**.

### 3.9 Thematic map

Through the keywords co-occurrence network, we could know the general research theme categories in this field. However, it was still difficult to pick which research direction should pursue in the future. Therefore, the “Thematic Map” module of the Bibliometric package was further applied to assist decision-making.

**Figure 8:**
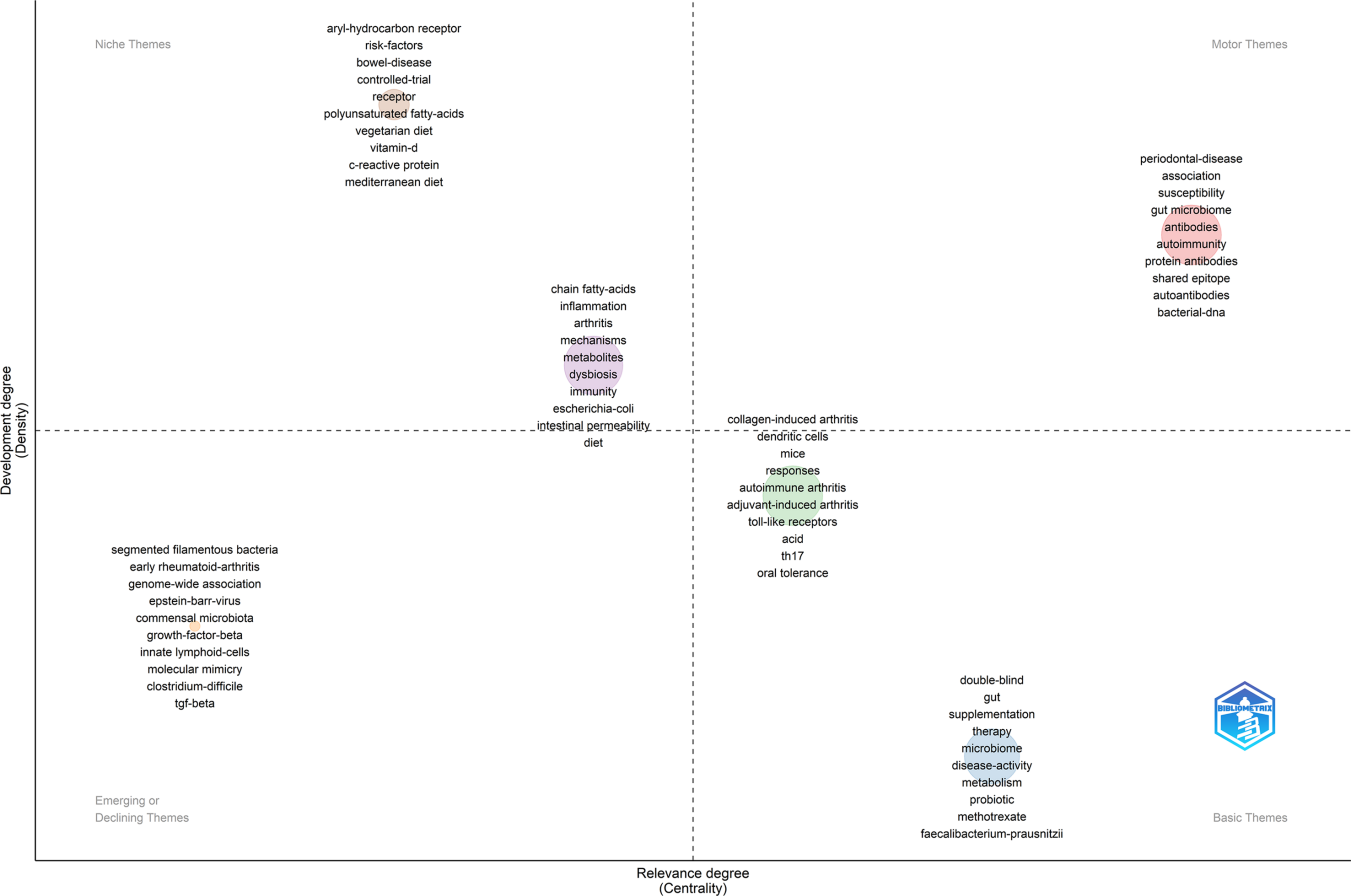
The thematic map of the field of GM in RA.

In the thematic map, the horizontal axis represented the centrality, which meant the relevance degree of the theme to this field. The vertical axis represented the density, which meant the development degree of the theme in this field. Four quadrants were drawn accordingly. The theme in the first quadrant (Motor Themes) was both important and well-developed. It could be seen from **Figure 8** that this quadrant contained keywords such as “association”, “gut microbiome”, “antibodies”, “autoimmune”, “protein antibodies”, “shared epitope”, “autoantibodies”, “bacterial-dna”. These keywords were partially coincident with those in Cluster 1 of the keywords co-occurrence network, mainly discussing the association between GM and RA. It told us that there was ample evidence to prove the close association between GM and RA, as well as the immune system, which was the cornerstone of research in this field.

The theme in the second quadrant (Niche Themes) was well-developed but not much relevant to the current field. It mainly contained two themes as **Figure 8**. One theme contained keywords “aryl-hydrocarbon receptor”, “risk-factors”, “bowel-disease”, “controlled-trial”, “receptor”, “polyunsaturated fatty-acids”, “vegetarian diet”, “vitamin-d”, “c-reactive protein” and “mediterranean diet”, and the development degree was larger but less central. It showed that a lot of studies have been carried out on eating habits affecting RA and GM. Another theme had better centrality, including keywords “chain fatty-acids”, “inflammation”, “mechanisms”, “metabolites”, “dysbiosis”, “immunity”, “escherichia-coli”, “intestinal permeability” and “diet”. It could be seen that the studies on the mechanism of GM participating in RA from the perspective of metabolite analysis have made decent progress.

The theme in the third quadrant (Emerging or Declining Themes) was not well-developed, just now emerging or about to disappear. It could be seen from **Figure 8** that the theme of this quadrant also belonged to mechanism research, which mainly focused on more targeted studies on the impact of some microorganisms such as “segmented filamentous bacteria” and “clostridium-difficile” on RA, and the role of some immune cells or immune factors such as “growth-factor-beta”, “innate lymphoid-cells” and “tgf-beta” in GM participating in RA.

The theme in the fourth quadrant (Basic Themes) was important but not well-developed in the field. One of the themes included keywords “double-blind”, “supplementation”, “therapy”, “metabolism”, “probiotic” and so on, concerning the studies on regulating GM to remedy RA. Another theme has better development and was mainly related to experimental research and important mechanism research, such as “th17” and “toll-like receptors”.

## 4 Discussion

### 4.1 Overview of development in the field of GM in RA

In this study, we retrieved 255 original research articles and 204 reviews related to the involvement of GM in RA from 2004 to 2022 in WOSCC. 2013 was the turning point of the annual growth of publications and the annual total citations, while 2018 was the vital time for further growth. These were two important periods. A large number of researchers have devoted themselves to this field (**Figure 4**), and many journals have begun to pay attention to it at this time (**Figure 5**). This might be related to the development and popularization of metanomic and metatranscriptomic tools, such as 16S rRNA gene pyrosequencing, metagenomic shotgun sequencing and cDNA sequencing, which made it more in-depth and convenient to carry out the research on the multi-species system of GM.

China and the United States had the deepest academic accumulation and the greatest influence in this field with the highest number of publications and citations. The affiliations with the largest contribution were also those of the United States and China. Interestingly, although the number of articles in China was slightly more than that in the United States, the number of citations was less than half of that in the United States, which should be due to the difference in the length of their research in this field. As can be seen from **Figure 2D**, the average output time of articles in China was later than that of the United States. It demonstrated that although the output of China was growing rapidly, the depth, breadth and accuracy of academic research in this field in the United States were far greater than that of China, which has newly joined this field. China and the United States also played a leading role in cooperation in this field (**Figures 3B**, **2D**).

About two-thirds of the affiliations only had one article. It indicated that most affiliations in this field had not made an in-depth investment, mainly a few affiliations have carried out continuous research.

The number of publications and the total citations can help objectively analyze the authors with the highest contribution and influence in this field. Scher, Jose U. from New York University in the USA and Taneja, Veena from Mayo Clinic in the USA undoubtedly had the greatest contribution in this field according to their most prolific output and citations. Although China had the largest number of outputs, few of the top-contributed authors were from China. Considering the late start of China, this suggested that there were a large number of researchers who have just invested in this field in China. They have contributed a lot of output but were generally not abundant for their personal accumulation.

There were many journals concerned with this field. The development of *Frontier* serial journals in this field showed vigorous momentum. In particular, although *Frontiers in Immunology* participated since 2017, its excellent annual growth made it the most productive. Notably, of the top-productive 10 and top-cited 10 journals, all but two had high a IF greater than 5.000. This meant that it was not a challenge to publish research on the GM in RA in high-quality journals.

In original research articles, “Zhang X, 2015, Nat Med” and “Scher JU, 2013, eLife” had the highest LCS. Similarly, the two articles mainly compared the GM differences between RA patients and healthy people. Among them, “Zhang X, 2015, Nat Med” reported that the intestinal flora of RA patients had dysbiosis, which could be alleviated after treatment with disease-modifying antirheumatic drugs (DMARDs). The metagenomic shotgun sequencing and a metagenome-wide association study (MGWAS) were used. “Scher JU, 2013, eLife” found that *Prevotella Copri* was closely related to RA by 16S sequencing and shotgun sequencing on feces samples from RA patients and healthy people, and they identified the potential role for *P. copri* in the pathogenesis of RA by fecal colonization experiment in mice. The top 10 highly local-cited original research articles mainly focused on the difference in GM between RA patients and healthy people, and the role of GM in the pathogenesis of RA. These articles provided important evidence for the relationship between GM and RA, and the applied technologies and research methods also gave references for relevant research.

In reviews, “Scher JU, 2011, Nat Rev Rheumatol”, “Brusca SB, 2014, Curr Opin Rheumatol” and “Horta-Baas G, 2017, J Immunol Res” received the most attention. These three articles have reviewed the link between microorganisms and RA, but the emphasis was different. “Scher JU, 2011, Nat Rev Rheumatol” was the highest cited review in this field and summarized the historical clues of the role of microorganisms in rheumatoid arthritis. In this review, Scher JU and Abramson SB combed the origin and paleopathology of RA and found that the concept of oral or GM associated with RA had emerged for more than a century. Moreover, from the relationship between microorganisms and their host to the relationship between microorganisms and the immune system, and finally to the relationship between microorganisms and RA pathogenesis, a series of possible evidence for microorganisms to participate in RA have been listed step by step in this review. It also pointed out that 16S rRNA pyrosequencing methods, shotgun analyses and gnotobiotic experiments were important methods for studying microorganisms and RA. “Brusca SB, 2014, Curr Opin Rheumatol” summarized the connection between mucosal microorganisms (the gut, the gingival, and the respiratory tree) and RA. “Horta-Baas G, 2017, J Immunol Res” combed the link between GM and RA from the perspective of autoimmune mechanism, in which the summary of relevant immune cells and immune factors was particularly detailed, and systematically sorted out the gut flora related to RA reported at that time. In these reviews, some drugs for RA that might be related to GM were also briefly introduced, such as sulfasalazine (SSZ). In the top 10 highly local-cited reviews, we can get a systematic understanding of the evidence of the association between GM and RA, and also get in-depth directions and results of researchers in this field.

### 4.2 Research structure, current status and future development trend of GM in RA

The keywords are a high summary of the theme and content of the literature. The frequency of keywords appearing in the literature of a field can reveal the research hotspots in this field. The frequency of two or more keywords appearing in the same literature can indicate the research structure of this field. Keywords co-occurrence analysis conducted by the two can indicate the research category and research hotspots in this field, and facilitate the discovery of new disciplinary growth points and trends. A systematic and general understanding of the research status in this field was presented to us after the analysis of keywords. However, more in-depth exploration is needed for the research details, which requires reading and sorting out the documents themselves. Therefore, keywords analysis and literature sorting were used to learn the research structure, current status and future development trend.

**Figure 9:**
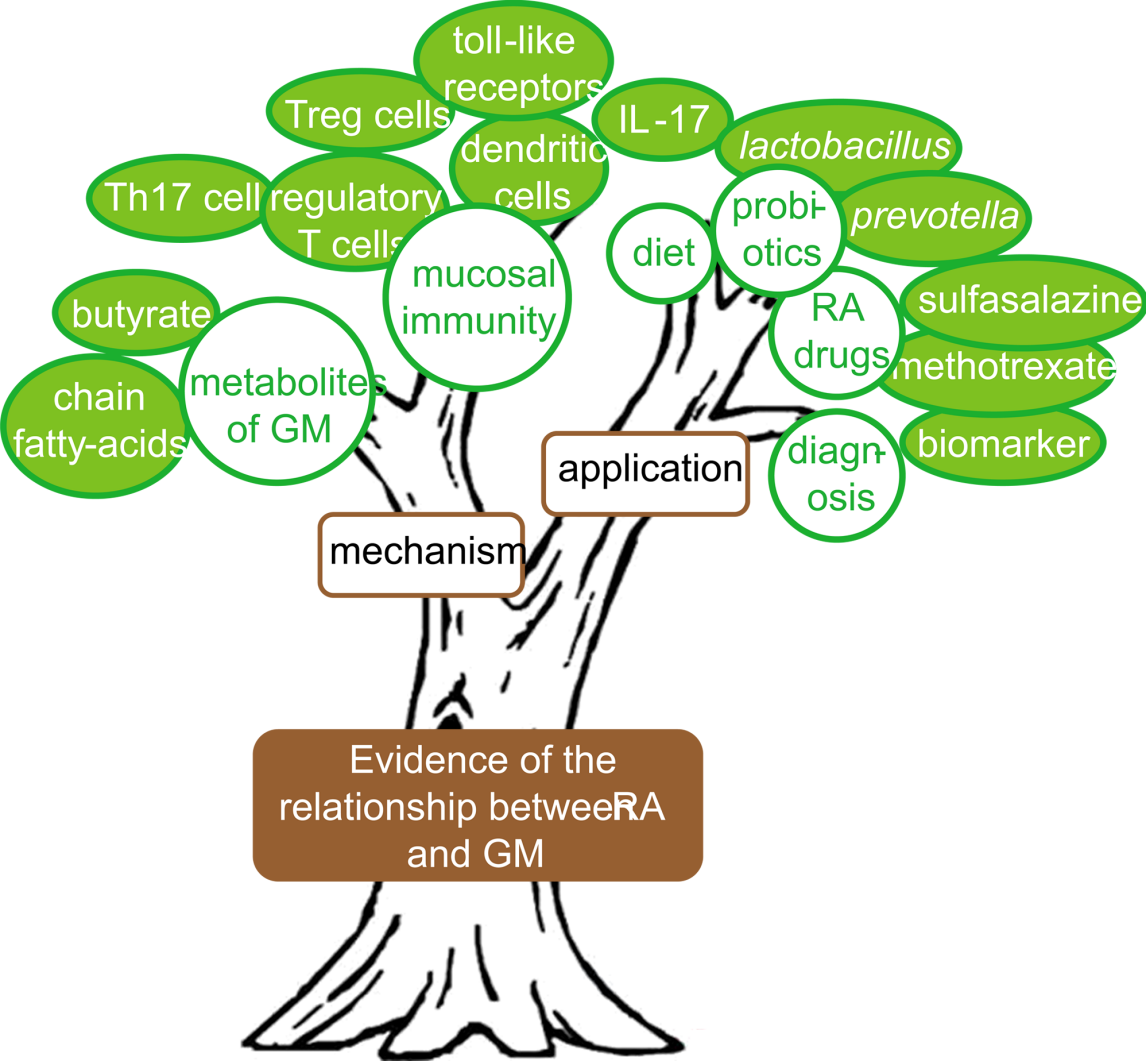
Research structure tree summarized from keywords analysis.

From the keywords co-occurrence network according to the time stamp (**Figure 7B**) and the changes of high-frequency keywords over the years (**Figure 7C**), it could be seen that although the studies on GM participating in RA have not been officially carried out for a long time, researchers have begun to actively explore in multiple directions. Especially since 2013, the research boom has begun and continues to this day. The research structure in this field could be summarized through the keywords co-occurrence network (**Figure 6**), as shown in **Figure 9**. The first part was to find evidence that RA is related to GM, which was the trunk. Then there was the mechanism research, in which there were several research branches, such as “th17” and “toll-like receptors”, “chain fatty-acids”, etc. In addition, there was also practical application research, such as probiotics supplement, dietary intervention and drug targeting GM. The collected documents were carefully read to obtain the details of the current status of GM in RA.

#### 4.2.1 Relationship between RA and GM

The first is the evidence that RA is related to GM and its mechanism. The two are often inseparable and belong to the research on the relationship between RA and GM. Relevant reports on the interaction between RA and GM have been presented in “Interaction between RA and GM” sheet in the **Supplementary Material 1**. A wide variety of microorganisms exist in the gut of human beings and play a noteworthy role in human health. The dysbiotic GM occurs in RA individuals, characterized by the changed abundance of many bacteria compared with that of healthy individuals (11–13). Significant disorders of GM appeared before visible arthritis symptoms and continued to evolve in the course of the disease (14, 15), representing different gut flora profiles at separate disease stages (16). The dysbiotic gut microbiota could partially be recovered after RA treatment (17). The condition of RA will also be affected when confronted with disturbed GM. The gut bacterial DNA was observed in synovial samples from RA patients, which could directly affect the severity of arthritis (18). Through the case-control study, it is found that antibiotic prescriptions are associated with a higher risk of RA, which may be due to immunological responses induced by dysfunctional GM (19, 20). And this is further proved by experimental evidence that oral administration of antibiotic (enrofloxacin) would aggravate arthritis (21). SKG mice transplanted with gut microbiota from RA patients showed more severe arthritis and increased intestinal TH17 cells (12). A higher frequency of arthritis induction occurred in germ-free mice transplanted with the gut microbiota of CIA-susceptible mice instead of CIA-resident mice (22). These tell us that the relationship between GM and RA is not unidirectional but bidirectional.

Among the diverse microbiota, which is close to RA and plays what role in the disease course? We have summarized the detected microbes in human intestines that may be related to RA, see **Supplementary Material 2**. Most bacteria in the human gut belong to the phylum *Firmicutes* and *Bacteroidetes*. The genera *Bacteroides* and *Prevotella* play an important role in the balance of gut microbiota as the dominant bacteria of *Bacteroides*. The abundance of *Prevotella* significantly increased in RA patients generally, whether before or after clinical diagnosis (12, 23, 24). Most species of *Prevotella* are generally considered to be pathogenic to RA and will promote inflammation. *Prevotella* has an association with the genotype of RA, that is, the high-risk individuals with RA (25). The enrichment of *Prevotella* in the feces of pre-clinical RA individuals further confirms it (23). *P. copri*, the most widely known species in *Prevotella*, could develop severe arthritis and induce the proinflammatory reaction of intestinal Th17 cells when it is colonized to mice (12, 26). But not all species in *Prevotella* are pathogenic to RA, for example, *P. histicola* is a therapeutic bacterium, which could reduce the incidence and severity of arthritis significantly and restore the microbial profile and metabolites of arthritis mice through the regulation of the CD1031 dendritic cells and myeloid suppressors (CD11b+Gr-1+cells), production of intestinal Treg cells and the further inhibition of antigen-specific Th17 responses and increase of transcription of IL-10 (27, 28). The alteration of the abundance of *Bacteroides* is not so consistent in different RA patients, and some are increasing (29–31), some are decreasing (32, 33). In fact, its role in immune function needs to be viewed in two ways (34). For instance, lipopolysaccharide (LPS) of *B. fragilis* significantly suppressed arthritis development in CIA model animals (35), while its strong virulence may destroy the immune balance of the host (34). The specialized research on the relationship between the bacteria in the genus *Bacteroides* and RA is unexpectedly few at present, so it can be further excavated.

In the phylum *Firmicutes*, several genera such as *Lactobacillus*, *Faecalibacterium*, *Streptococcus*, *Blautia* have been proven to be related to RA. Among them, *Lactobacillus*, exiting not only in the gut but also in many fermented foods, receives a lot of attention. Many species of *Lactobacillus* can attenuate arthritis and inflammation, examples illustrating as *L. reuteri*, *L. casei* (36–38), *L. rhamnosus*, *L. acidophilus* (39, 40), *L. brevis*, *L. salivarius* (41) and *L. fermentum* (42). These strains of *Lactobacillus* protected against RA by rebalancing the GM and metabolites (short-chain fatty acids, SCFAs). Their therapeutic activity may involve different pathways, give examples, *L. salivarius* and *L. plantarum* reduce the Th17 cell fraction and increase the Treg fraction (41), *L. reuteri* and *L. casei* debilitate Th1 immune response, while *L. rhamnosus* and *L. fermentum* impair Th17 responses (42). The immunomodulatory properties of *Lactobacillus* depend on the strains and prebiotics (42, 43). It can be seen that most species of *Lactobacillus* play the role of beneficial bacteria in RA. It is generally believed that the abundance of beneficial bacteria is reduced while that of pathogenic bacteria is increased in the intestinal bacteria of diseased individuals. This is consistent with the decrease of *Lactobacillus* abundance in most RA patients and arthritis animal models (22, 32, 44, 45). However, there are exceptions, Xuan Zhang et al. (17) identified the gut, dental and saliva microbiome of RA patients, and the results showed that *L. salivarius* was over-represented on these three sides. Enrichment of *Lactobacillus* observed in RA patients was reported by Yumei Chen et al. (30) and Liu, XF et al. (46). Different manifestations of *Lactobacillus* in RA patients may be a consequence of RA progression and the different conditions of subjects.

In addition to the bacteria mentioned above, there are other ones whose functions on RA have been explored. *Bifidobacterium longum* RAPO could significantly reduce RA incidence, arthritis and bone damage by inhibiting the production of IL-17 and other proinflammatory mediators (47). *Bifidobacterium* sp. decreased the production of pro-inflammatory monocyte chemotactic protein-1 (MCP-1) and MCP-3 and the number of inflammatory monocytes CD11c+Ly6c+ (48). But the function of another species *B. adolescentis* in the genus *Bifidobacterium* showed a contradiction in different experiments. The colonization with *B. adolescentis* in K/BxN mouse model could induce Th17 cells in the murine intestine and exacerbate autoimmune arthritis just like segmented filamentous bacteria (49). Early *B. adolescentis* administration before modelling reduced the clinical symptoms, maintained the fecal concentration of SCFAs, as well as restoring the intestinal dysbiosis in CIA rats, which performed better than late-treatment (50). The opposite functional performance of *B. adolescentis* may be due to the differences in research objects or disease stages, or other factors, which needs further research to explain. *Clostridioides. difficile* VPI10463 could reduce the incidence of CIA, but its intervention after the establishment of CIA has weakened therapeutic effect (51). *Eggerthella lenta* and *Collinsella aerofaciens* could enhance gut permeability (48). Mice treated with *E. lenta* increased Th17 cytokines and the arthritis was augmented in CIA model mice gavaged with *C. aerofaciens*. *Candida albicans* also plays an unfavorable role that making arthritis heavier (52). It is worth mentioning that the highly concerned oral bacteria, *Porphyromonas gingivalis*, could also cause intestinal flora dysbiosis, improve the Th17 cell proportions in mesenteric lymphocytes and the level of citrullinated protein and IL-6, and then aggravate arthritis (53, 54).

Besides, some researchers explored the intervention effect of several mixed bacteria on RA such as probiotics supplements. The mixture of *Lactobacillus acidophilus*, *L. casei*, *L. lactis*, *Bifidobacterium lactis* and *B. bifidum* could reduce the inflammatory biomarkers and improve the oxidative/nitrosative profile in RA patients (40). *Bifidobacterium* triple viable (*Bifidobacterium*, *Lactobacillus* and *Enterococcus*) could partially reverse the gut flora and serum inflammation in rats with RA (39).

At present, the mechanisms of GM participating in RA pathogenesis mainly include regulating the differentiation of immune cells, inducing the production of inflammatory mediators, and molecular simulation. Disturbed GM can trigger the innate and adaptive immunity abnormally, which may lead to aberrant systemic immunity (55–57). The mucosal immunity is the first line of defense against exogenous pathogens in the gastrointestinal tract (58, 59). The GM can regulate the activation of innate immune cells, causing the release of pro-inflammatory or anti-inflammatory cytokines such as IL-6, TNF-α, IL-1β, IL-12 and IL-23 (6, 60). The differentiation of T cells and B cells is affected by GM and then instigates RA, especially Th17 and Treg cells (22). IL-17 secreted by Th17 cells can promote RA (61). Conversely, IL-10 and TGF-β1 secreted by Treg cells can inhibit RA and inhibit Th17 cells (62, 63). Th17/Treg balance is closely related to RA and strongly affected by GM (64). Human leukocyte antigen (HLA) may be directly or indirectly involved (65). Autoantibodies and cytokines produced by immune cells are transported to tissues and organs through circulation to activate macrophages, which leads to the release of pro-inflammatory factors (60). In addition, the GM can cross the damaged intestinal barrier and migrate to the tissues, triggering autoimmune inflammation and then increasing the risk of RA (11, 18). The epitopes of intestinal microbial proteins homologous to self-peptides may trigger autoimmunity, which is called molecular mimicry (66). The GM may participate in the pathogenesis of RA through molecular mimicry. For instance, the HLA-DR-presented N-acetylglucosamine-6-sulfatase share sequence homology with sulfatase proteins of *Parabacteroides* sp. and *Prevotella* sp., and the HLA-DR-presented filamin A is also homologous with epitopes from proteins of the *Butyricimonas* sp. and *Prevotella* sp. (67). Aside above mechanisms, the GM may affect RA by regulating the level of sex hormone levels, protein citrullination, etc. (68, 69). In these processes, the metabolites of GM, such as short-chain fatty acids, bile acids and tryptophan metabolites, play a bridge role in the immune response triggered by GM (57). However, most of the details in these mechanisms are not clear.

Due to the abundance and diversity of intestinal microorganisms and the complex pathological mechanism of RA, the research work in this field is not easy to carry forward. Fortunately, with the development of science and technology, 16S rRNA gene sequencing, metagenome sequencing, gene chip, fluorescence *in situ* hybridization, etc. have become fashionable. In particular, 16S rRNA gene sequencing has become a mainstream bacterial identification technology, which helps researchers have a better understanding of the complex composition of gut microbiota. Fecal microbiota transplantation (FMT)/colonization is also effective means to help observe the influence of flora on the host.

Obviously, evidence is sufficient to prove that there is a correlation between GM and RA, but many details are unclear at present. Some bacteria have been reported to have significant differences between healthy and RA individuals, or affect immune function, or be related to other autoimmune diseases, but there is no specific report on RA, which is not much and deep-going (5, 6, 70). FMT/colonization is a method worth considering, but it is not feasible in all cases. For example, the dysbiotic gut microbiota of TNF^ΔARE+/−^ mice could be transferred to germ-free mice but not to conventional healthy mice (71). This suggests that we should carefully design and choose the experiment according to the purpose. In addition, the GM is such a colorful composition that they usually act together rather than alone. However, it is not clear which microorganisms interact with each other and what details are involved. There are few reports in this regard. In the mechanism study, the researchers have gone through different tracks, but it is so complicated that it has only shown only a speck in a vast ocean from current reports. It can be seen that this field has already had a solid foundation, but there is still broad space to explore in the future.

#### 4.2.2 Application potential of GM in RA

There is no doubt that the GM is closely related to RA, which is a worthy way to explore for the diagnosis and treatment of RA, and relevant reports have been presented in “Application of GM in RA” sheet in the **Supplementary Material 1**. The alternation of GM can be applied in the prediction and treatment of RA. Before the onset of RA, there are some presages about the high risk of the disease such as rheumatoid factor (RF) and anti-citrullinated protein (anti-CCP) (23, 72). The gut microbiome of Anti-CCP/RF positive high-risk individuals without clinical synovitis differs significantly from that of healthy individuals. The study of SCREEN-RA cohort also uncovered gut microbiome as a risk factor for RA development (73). This can help discover new biological markers of progression toward RA, which is beneficial to identify high-risk populations precisely.

In addition, there is a connection between the gut microbial signatures and the trajectory of disease activity in RA (74), so there are potential therapeutic targets to be dug. A lot of work has been carried out and particularly some researchers have introduced *in silico* methods such as data mining and machine learning algorithms to analyze clinical data, microbiome and multiomics data, so as to discover biomarkers and pathways, and then help explore key bacteria and mechanisms (74–78). However, many results from *in silico* research need further verification.

Interventions to prevent/treat RA through GM mainly include probiotics and their metabolites, diet and RA drugs. Probiotics such as *Lactobacillus* strains, *Bifidobacterium* spp. and their metabolites such as SCFAs (especially butyrate) display a great development prospect (79–81). Fasting therapy and high fiber diet can help alleviate RA and can be used as auxiliary treatment, while the Mediterranean diet aggravates arthritis (82–84). In addition, researchers also pay attention to the application of GM in existing RA drugs. For example, It has been reported that the response of RA patients to methotrexate (MTX) is related to the gut microbiota profiles (85, 86), which can help the better clinical application of MTX. However, the role of MTX in intestinal bacteria is not beneficial. MTX will cause gut microbiota disorder in RA patients, and affect the conserved pathways of many gut bacteria to reduce immune activation (87, 88). This adverse effect on gut microbiota can be compensated by probiotics or propionate (88, 89). SSZ has an antibacterial effect, and its metabolism depends on azoreductase (AR). Probiotic strains possess AR activity and can metabolize SSZ but have no effect on plasma levels of SSZ and sulfapyridine (SP) (90). Etanercept (ETN), a TNF-α antagonist, can partially restore a beneficial microbiota of RA patients and alter gut microbiota in CIA mice (91, 92). It is worth noting that many traditional medicines, such as traditional Chinese medicine (TCM), have also shown their regulatory effects on gut microbiota (seen in “Application of GM in RA” sheet in **Supplementary Material 1**). The properties of multi-component and multi-target, and oral administration as the common administration mode of TCM stimulate researchers to pay attention to this field. It can be seen that researchers are actively exploring the application of GM in the treatment of RA. However, it is restricted that most related studies are efficacy studies plus microbiota identification, which is still on the surface and can only tell us that the treatment of these objects will affect the composition of gut microbiota. The role of microbial regulation in drug efficacy is still mysterious, let alone its internal mechanism.

#### 4.2.3 Future development trend

It can be seen from “Thematic Map” (**Figure 8**) that there were abundant studies to reveal the relationship between GM and RA so as to build a solid foundation for this field. But it was still at the superficial level at present, and the deeper and more detailed mechanism exploration has just begun to develop and is insufficient. These were consistent with the results of literature sorting. First, the effect of most bacterial strains on RA was still unknown. Only a small amount strains had special reports on RA. It is generally believed that in disease condition the beneficial bacteria will decrease and the pathogenic bacteria will increase, but not without exception, such as *L. Salivarius*, whose reason is still unknown. Even some bacteria have contradictory effects on RA in current reports (*B. adolescentis*). In addition, the internal mechanisms insight to causality are still inadequate. Some progress has been made in tracks such as TH17 and Treg cells, toll-like receptors, chain fatty-acids, etc., but further research is needed. It indicates that more specialized research and data are needed to clarify the role of GM in RA. Gratifyingly, the applications in this field have also developed rapidly and shown desirable potential. Omics technology, machine learning technology and animal models also provide opportunities for in-depth exploration of mechanisms. Therefore, researchers should pay more attention to the in-depth mechanism, which is a broad space to explore.

## 5 Conclusion

Based on the review and original articles from WOSCC in this field, we got a comprehensive and systematic understanding of the research field of GM in RA through bibliometrics. Output in this field was growing vigorously, especially in the past decade. More and more researchers and affiliations in different countries/regions have joined this field, and the increasing number of academic journals have paid attention to it. China and the United States played the most active roles. Recently, the mechanism and application of GM participating in RA have become hotspots of researches. There has been abundant evidence from research on humans and animals that RA and GM have a mutual influence on each other. However, the complicated details in it have not been explained clearly. In a word, this field has already established a solid foundation, and more efforts are needed to build it higher so as to seek new strategies for the treatment of RA patients.

## Data availability statement

The original contributions presented in the study are publicly available. This data can be found here: https://doi.org/10.6084/m9.figshare.c.6381747.v1.

## Author contributions

YD: conceptualization, formal analysis, and writing - original draft. JY: validation. QD: investigation and resources. XL: visualization. YH: visualization. XR: data curation. YZ: data curation. RS: investigation. XZ: investigation. JM: writing - review & editing. DS: investigation. FL: investigation. XW: writing - review & editing. RY: writing - review & editing and project administration. GS: writing - review & editing and conceptualization. All authors contributed to the article and approved the submitted version.

## References

[1] Burmester GR Pope JE. Novel treatment strategies in rheumatoid arthritis. Lancet (2017) 389(10086):2338–48. doi: 10.1016/S0140-6736(17)31491-5 28612748

[2] Catrina AI Deane KD Scher JU. Gene, environment, microbiome and mucosal immune tolerance in rheumatoid arthritis. Rheumatology (2016) 55(3):391–402. doi: 10.1093/rheumatology/keu469 25539828 PMC4746430

[3] Qin J Li R Raes J Arumugam M Burgdorf KS Manichanh C. A human gut microbial gene catalogue established by metagenomic sequencing. Nature (2010) 464(7285):59–65. doi: 10.1038/nature08821 20203603 PMC3779803

[4] Scher JU Abramson SB. The microbiome and rheumatoid arthritis. Nat Rev Rheumatol (2011) 7(10):569–78. doi: 10.1038/nrrheum.2011.121 PMC3275101 21862983

[5] Brusca SB Abramson SB Scher JU. Microbiome and mucosal inflammation as extra-articular triggers for rheumatoid arthritis and autoimmunity. Curr Opin Rheumatol (2014) 26(1):101–7. doi: 10.1097/BOR.0000000000000008 PMC4011633 24247114

[6] Horta-Baas G Romero-Figueroa MD Montiel-Jarquin AJ Pizano-Zarate ML Garcia-Mena J Ramirez-Duran N. Intestinal dysbiosis and rheumatoid arthritis: A link between gut microbiota and the pathogenesis of rheumatoid arthritis. J Immunol Res (2017) 2017:13. doi: 10.1155/2017/4835189 PMC5602494 28948174

[7] Moral-Muñoz JA Herrera-Viedma E Santisteban-Espejo A Cobo MJ. Software tools for conducting bibliometric analysis in science: An up-to-date review. El Profesional la Información. (2020) 29(1):e290103. doi: 10.3145/epi.2020.ene.03

[8] Xu P Lv T Dong S Cui Z Luo X Jia B. Association between intestinal microbiome and inflammatory bowel disease: Insights from bibliometric analysis. Comput Struct Biotechnol J (2022) 20:1716–25. doi: 10.1016/j.csbj.2022.04.006 PMC9019919 35495114

[9] van Eck NJ Waltman L. Software survey: VOSviewer, a computer program for bibliometric mapping. Scientometrics (2010) 84(2):523–38. doi: 10.1007/s11192-009-0146-3 PMC2883932 20585380

[10] Aria M Cuccurullo C. Bibliometrix: An r-tool for comprehensive science mapping analysis. J Informetrics. (2017) 11(4):959–75. doi: 10.1016/j.joi.2017.08.007

[11] Chen J Wright K Davis JM Jeraldo P Marietta EV Murray J. An expansion of rare lineage intestinal microbes characterizes rheumatoid arthritis. Genome Med (2016) 8(1):43. doi: 10.1186/s13073-016-0299-7 27102666 PMC4840970

[12] Maeda Y Kurakawa T Umemoto E Motooka D Ito Y Gotoh K. Dysbiosis contributes to arthritis development *via* activation of autoreactive T cells in the intestine. Arthritis Rheumatol (2016) 68(11):2646–61. doi: 10.1002/art.39783 27333153

[13] Yu D Du J Pu X Zheng L Chen S Wang N. The gut microbiome and metabolites are altered and interrelated in patients with rheumatoid arthritis. Front Cell Infect Microbiol (2021) 11:763507. doi: 10.3389/fcimb.2021.763507 35145919 PMC8821809

[14] Jubair WK Hendrickson JD Severs EL Schulz HM Adhikari S Ir D. Modulation of inflammatory arthritis in mice by gut microbiota through mucosal inflammation and autoantibody generation. Arthritis Rheumatol (2018) 70(8):1220–33. doi: 10.1002/art.40490 PMC6105374 29534332

[15] Rogier R Evans-Marin H Manasson J van der Kraan PM Walgreen B Helsen MM. Alteration of the intestinal microbiome characterizes preclinical inflammatory arthritis in mice and its modulation attenuates established arthritis. Sci Rep (2017) 7(1):15613. doi: 10.1038/s41598-017-15802-x 29142301 PMC5688157

[16] Nemoto N Takeda Y Nara H Araki A Gazi MY Takakubo Y. Analysis of intestinal immunity and flora in a collagen-induced mouse arthritis model: Differences during arthritis progression. Int Immunol (2020) 32(1):49–56. doi: 10.1093/intimm/dxz058 31562738

[17] Zhang X Zhang D Jia H Feng Q Wang D Liang D. The oral and gut microbiomes are perturbed in rheumatoid arthritis and partly normalized after treatment. Nat Med (2015) 21(8):895–905. doi: 10.1038/nm.3914 26214836

[18] Siala M Jaulhac B Gdoura R Sibilia J Fourati H Younes M. Analysis of bacterial DNA in synovial tissue of Tunisian patients with reactive and undifferentiated arthritis by broad-range PCR, cloning and sequencing. Arthritis Res Ther (2008) 10(2):R40. doi: 10.1186/ar2398 18412942 PMC2453759

[19] Sultan AA Mallen C Muller S Hider S Scott I Helliwell T. Antibiotic use and the risk of rheumatoid arthritis: A population-based case-control study. BMC Med (2019) 17(1):154. doi: 10.1186/s12916-019-1394-6 31387605 PMC6685281

[20] Armstrong D Dregan A Ashworth M White P McGee C de Lusignan S. Influence of prior antibiotic use on risk of rheumatoid arthritis: Case control study in general practice. Rheumatol (Oxford). (2020) 59(6):1281–7. doi: 10.1093/rheumatology/kez452 31580454

[21] Dorozynska I Majewska-Szczepanik M Marcinska K Szczepanik M. Partial depletion of natural gut flora by antibiotic aggravates collagen induced arthritis (CIA) in mice. Pharmacol Rep (2014) 66(2):250–5. doi: 10.1016/j.pharep.2013.09.007 24911078

[22] Liu X Zeng B Zhang J Li W Mou F Wang H. Role of the gut microbiome in modulating arthritis progression in mice. Sci Rep (2016) 6:30594. doi: 10.1038/srep30594 27481047 PMC4969881

[23] Alpizar-Rodriguez D Lesker TR Gronow A Gilbert B Raemy E Lamacchia C. Prevotella copri in individuals at risk for rheumatoid arthritis. Ann Rheum Dis (2019) 78(5):590–3. doi: 10.1136/annrheumdis-2018-214514 30760471

[24] Kishikawa T Maeda Y Nii T Motooka D Matsumoto Y Matsushita M. Metagenome-wide association study of gut microbiome revealed novel aetiology of rheumatoid arthritis in the Japanese population. Ann Rheum Dis (2020) 79(1):103–11. doi: 10.1136/annrheumdis-2019-215743 PMC6937407 31699813

[25] Wells PM Adebayo AS Bowyer RCE Freidin MB Finckh A Strowig T. Associations between gut microbiota and genetic risk for rheumatoid arthritis in the absence of disease: A cross-sectional study. Lancet Rheumatol (2020) 2(7):e418–e27. doi: 10.1016/S2665-9913(20)30064-3 PMC7729822 33345197

[26] Jiang L Shang M Yu S Liu Y Zhang H Zhou Y. A high-fiber diet synergizes with prevotella copri and exacerbates rheumatoid arthritis. Cell Mol Immunol (2022) 19(12):1414–24. doi: 10.1038/s41423-022-00934-6 PMC9709035 36323929

[27] Marietta EV Murray JA Luckey DH Jeraldo PR Lamba A Patel R. Suppression of inflammatory arthritis by human gut-derived prevotella histicola in humanized mice. Arthritis Rheumatol (2016) 68(12):2878–88. doi: 10.1002/art.39785 PMC5125894 27337150

[28] Balakrishnan B Luckey D Bodhke R Chen J Marietta E Jeraldo P. Prevotella histicola protects from arthritis by expansion of allobaculum and augmenting butyrate production in humanized mice. Front Immunol (2021) 12:609644. doi: 10.3389/fimmu.2021.609644 34017324 PMC8130672

[29] Scher JU Sczesnak A Longman RS Segata N Ubeda C Bielski C. Expansion of intestinal prevotella copri correlates with enhanced susceptibility to arthritis. Elife (2013) 2:e01202. doi: 10.7554/eLife.01202 24192039 PMC3816614

[30] Chen YM Ma CY Liu LX He JQ Zhu CX Zheng FP. Analysis of gut microbiota and metabolites in patients with rheumatoid arthritis and identification of potential biomarkers. Aging-US (2021) 13(20):23689–701. doi: 10.18632/aging.203641 PMC8580343 34670873

[31] Wang Q Zhang SX Chang MJ Qiao J Wang CH Li XF. Characteristics of the gut microbiome and its relationship with peripheral CD4(+) T cell subpopulations and cytokines in rheumatoid arthritis. Front Microbiol (2022) 13:799602. doi: 10.3389/fmicb.2022.799602 35185845 PMC8851473

[32] Sun Y Chen Q Lin P Xu R He D Ji W. Characteristics of gut microbiota in patients with rheumatoid arthritis in shanghai, China. Front Cell Infect Microbiol (2019) 9:369. doi: 10.3389/fcimb.2019.00369 31709198 PMC6819506

[33] El Menofy NG Ramadan M Abdelbary ER Ibrahim HG Azzam AI Ghit MM. Bacterial compositional shifts of gut microbiomes in patients with rheumatoid arthritis in association with disease activity. Microorganisms (2022) 10(9):1820. doi: 10.3390/microorganisms10091820 36144422 PMC9505928

[34] Wexler HM. Bacteroides: The good, the bad, and the nitty-gritty. Clin Microbiol Rev (2007) 20(4):593–621. doi: 10.1128/CMR.00008-07 17934076 PMC2176045

[35] Kitamura K Sasaki M Matsumoto M Shionoya H Iida K. Protective effect of bacteroides fragilis LPS on escherichia coli LPS-induced inflammatory changes in human monocytic cells and in a rheumatoid arthritis mouse model. Immunol Lett (2021) 233:48–56. doi: 10.1016/j.imlet.2021.03.008 33741378

[36] Pan H Guo R Ju Y Wang Q Zhu J Xie Y. A single bacterium restores the microbiome dysbiosis to protect bones from destruction in a rat model of rheumatoid arthritis. Microbiome (2019) 7(1):107. doi: 10.1186/s40168-019-0719-1 31315667 PMC6637628

[37] Fan Z Ross RP Stanton C Hou B Zhao J Zhang H. Lactobacillus casei CCFM1074 alleviates collagen-induced arthritis in rats *via* balancing Treg/Th17 and modulating the metabolites and gut microbiota. Front Immunol (2021) 12:680073. doi: 10.3389/fimmu.2021.680073 34079556 PMC8165437

[38] Vaghef-Mehrabany E Alipour B Homayouni-Rad A Sharif SK Asghari-Jafarabadi M Zavvari S. Probiotic supplementation improves inflammatory status in patients with rheumatoid arthritis. Nutrition (2014) 30(4):430–5. doi: 10.1016/j.nut.2013.09.007 24355439

[39] Jin ZL Chen XC. Changes in intestinal florae and serum inflammation in rheumatoid arthritis rats and the effects of probiotics. Eur Rev Med Pharmacol Sci (2020) 24(22):11820–6. doi: 10.26355/eurrev_202011_23839 33275254

[40] Cannarella LAT Mari NL Alcantara CC Iryioda TMV Costa NT Oliveira SR. Mixture of probiotics reduces inflammatory biomarkers and improves the oxidative/nitrosative profile in people with rheumatoid arthritis. Nutrition (2021) 89:111282. doi: 10.1016/j.nut.2021.111282 34111674

[41] Liu X Zhang J Zou Q Zhong B Wang H Mou F. Lactobacillus salivarius isolated from patients with rheumatoid arthritis suppresses collagen-induced arthritis and increases treg frequency in mice. J Interferon Cytokine Res (2016) 36(12):706–12. doi: 10.1089/jir.2016.0057 27845855

[42] Fan Z Yang B Ross RP Stanton C Zhao J Zhang H. The prophylactic effects of different lactobacilli on collagen-induced arthritis in rats. Food Funct (2020) 11(4):3681–94. doi: 10.1039/C9FO02556A 32301444

[43] Sredkova P Batsalova T Moten D Dzhambazov B. Prebiotics can change immunomodulatory properties of probiotics. Cent Eur J Immunol (2020) 45(3):248–55. doi: 10.5114/ceji.2020.101237 PMC7790008 33437176

[44] Wang Y Li HB Wang J Zhao J Tie N Bai LJ. Associations of changes in serum inflammatory factors, MMP-3, 25(OH)D and intestinal flora with osteoporosis and disease activity in rheumatoid arthritis patients. Clin Lab (2020) 66(12):2443–8. doi: 10.7754/Clin.Lab.2020.200242 33337838

[45] Xu H Cao J Li X Lu X Xia Y Fan D. Regional differences in the gut microbiota and gut-associated immunologic factors in the ileum and cecum of rats with collagen-induced arthritis. Front Pharmacol (2020) 11:587534. doi: 10.3389/fphar.2020.587534 33442384 PMC7797777

[46] Liu X Zou Q Zeng B Fang Y Wei H. Analysis of fecal lactobacillus community structure in patients with early rheumatoid arthritis. Curr Microbiol (2013) 67(2):170–6. doi: 10.1007/s00284-013-0338-1 23483307

[47] Jeong Y Jhun J Lee SY Na HS Choi J Cho KH. Therapeutic potential of a novel bifidobacterium identified through microbiome profiling of RA patients with different RF levels. Front Immunol (2021) 12:736196. doi: 10.3389/fimmu.2021.736196 34867956 PMC8634832

[48] Balakrishnan B Luckey D Taneja V. Autoimmunity-associated gut commensals modulate gut permeability and immunity in humanized mice. Mil Med (2019) 184(Suppl 1):529–36. doi: 10.1093/milmed/usy309 30901468

[49] Tan TG Sefik E Geva-Zatorsky N Kua L Naskar D Teng F. Identifying species of symbiont bacteria from the human gut that, alone, can induce intestinal Th17 cells in mice. Proc Natl Acad Sci U S A. (2016) 113(50):E8141–E50. doi: 10.1073/pnas.1617460113 PMC5167147 27911839

[50] Fan Z Yang B Ross RP Stanton C Shi G Zhao J. Protective effects of bifidobacterium adolescentis on collagen-induced arthritis in rats depend on timing of administration. Food Funct (2020) 11(5):4499–511. doi: 10.1039/D0FO00077A 32383727

[51] Schmidt CJ Wenndorf K Ebbers M Volzke J Muller M Strubing J. Infection with clostridioides difficile attenuated collagen-induced arthritis in mice and involved mesenteric t(reg) and T(h2) polarization. Front Immunol (2020) 11:571049. doi: 10.3389/fimmu.2020.571049 33193352 PMC7662472

[52] Sonoyama K Miki A Sugita R Goto H Nakata M Yamaguchi N. Gut colonization by candida albicans aggravates inflammation in the gut and extra-gut tissues in mice. Med Mycol (2011) 49(3):237–47. doi: 10.3109/13693786.2010.511284 20807027

[53] Sato K Takahashi N Kato T Matsuda Y Yokoji M Yamada M. Aggravation of collagen-induced arthritis by orally administered porphyromonas gingivalis through modulation of the gut microbiota and gut immune system. Sci Rep (2017) 7(1):6955. doi: 10.1038/s41598-017-07196-7 28761156 PMC5537233

[54] Hamamoto Y Ouhara K Munenaga S Shoji M Ozawa T Hisatsune J. Effect of porphyromonas gingivalis infection on gut dysbiosis and resultant arthritis exacerbation in mouse model. Arthritis Res Ther (2020) 22(1):249. doi: 10.1186/s13075-020-02348-z 33076980 PMC7574451

[55] Zhao T Wei YY Zhu YY Xie ZH Hai QS Li ZF. Gut microbiota and rheumatoid arthritis: From pathogenesis to novel therapeutic opportunities. Front Immunol (2022) 13:8. doi: 10.3389/fimmu.2022.1007165 PMC9499173 36159786

[56] Opoku YK Asare KK Ghartey-Quansah G Afrifa J Bentsi-Enchill F Ofori EG. Intestinal microbiome-rheumatoid arthritis crosstalk: The therapeutic role of probiotics. Front Microbiol (2022) 13:7. doi: 10.3389/fmicb.2022.996031 PMC9623317 36329845

[57] Xu XY Wang M Wang ZK Chen Q Chen XX Xu YY. The bridge of the gut-joint axis: Gut microbial metabolites in rheumatoid arthritis. Front Immunol (2022) 13:14. doi: 10.3389/fimmu.2022.1007610 PMC9583880 36275747

[58] Matei DE Menon M Alber DG Smith AM Nedjat-Shokouhi B Fasano A. Intestinal barrier dysfunction plays an integral role in arthritis pathology and can be targeted to ameliorate disease. Med (N Y). (2021) 2(7):864–83 e9. doi: 10.1016/j.medj.2021.04.013 34296202 PMC8280953

[59] Tajik N Frech M Schulz O Schalter F Lucas S Azizov V. Targeting zonulin and intestinal epithelial barrier function to prevent onset of arthritis. Nat Commun (2020) 11(1):1995. doi: 10.1038/s41467-020-15831-7 32332732 PMC7181728

[60] Zhang X Chen BD Zhao LD Li H. The gut microbiota: Emerging evidence in autoimmune diseases. Trends Mol Med (2020) 26(9):862–73. doi: 10.1016/j.molmed.2020.04.001 32402849

[61] Kuwabara T Ishikawa F Kondo M Kakiuchi T. The role of IL-17 and related cytokines in inflammatory autoimmune diseases. Mediat Inflamm (2017) 2017:3908061. doi: 10.1155/2017/3908061 PMC5337858 28316374

[62] Xu H Zhao H Lu C Qiu Q Wang G Huang J. Triptolide inhibits osteoclast differentiation and bone resorption *In vitro via* enhancing the production of IL-10 and TGF-β1 by regulatory T cells. Mediat Inflammation (2016) 2016:8048170. doi: 10.1155/2016/8048170 PMC4930824 27413257

[63] Chen X Oppenheim JJ. Th17 cells and tregs: Unlikely allies. J Leukoc Biol (2014) 95(5):723–31. doi: 10.1189/jlb.1213633 PMC3984971 24563509

[64] Cheng H Guan X Chen D Ma W. The Th17/Treg cell balance: A gut microbiota-modulated story. Microorganisms (2019) 7(12):583. doi: 10.3390/microorganisms7120583 31756956 PMC6956175

[65] Gomez A Luckey D Yeoman CJ Marietta EV Berg Miller ME Murray JA. Loss of sex and age driven differences in the gut microbiome characterize arthritis-susceptible 0401 mice but not arthritis-resistant 0402 mice. PloS One (2012) 7(4):e36095. doi: 10.1371/journal.pone.0036095 22553482 PMC3338357

[66] Negi S Singh H Mukhopadhyay A. Gut bacterial peptides with autoimmunity potential as environmental trigger for late onset complex diseases: In-silico study. PloS One (2017) 12(7):e0180518. doi: 10.1371/journal.pone.0180518 28678867 PMC5498033

[67] Pianta A Arvikar SL Strle K Drouin EE Wang Q Costello CE. Two rheumatoid arthritis-specific autoantigens correlate microbial immunity with autoimmune responses in joints. J Clin Invest. (2017) 127(8):2946–56. doi: 10.1172/JCI93450 PMC5531397 28650341

[68] Ridlon JM Ikegawa S Alves JM Zhou B Kobayashi A Iida T. Clostridium scindens: A human gut microbe with a high potential to convert glucocorticoids into androgens. J Lipid Res (2013) 54(9):2437–49. doi: 10.1194/jlr.M038869 PMC3735941 23772041

[69] Li MX Wang F. Role of intestinal microbiota on gut homeostasis and rheumatoid arthritis. J Immunol Res (2021) 2021:9. doi: 10.1155/2021/8167283 PMC8203374 34195296

[70] Wu HJ Wu E. The role of gut microbiota in immune homeostasis and autoimmunity. Gut Microbes (2012) 3(1):4–14. doi: 10.4161/gmic.19320 22356853 PMC3337124

[71] Edwards V Smith DL Meylan F Tiffany L Poncet S Wu WW. Analyzing the role of gut microbiota on the onset of autoimmune diseases using TNF (DeltaARE) murine model. Microorganisms (2021) 10(1):73. doi: 10.3390/microorganisms10010073 35056521 PMC8779571

[72] Rooney CM Mankia K Mitra S Moura IB Emery P Wilcox MH. Perturbations of the gut microbiome in anti-CCP positive individuals at risk of developing rheumatoid arthritis. Rheumatol (Oxford). (2021) 60(7):3380–7. doi: 10.1093/rheumatology/keaa792 33313854

[73] Gilbert BTP Lamacchia C Mongin D Lauper K Trunk E Studer O. Cohort profile: SCREEN-RA: Design, methods and perspectives of a Swiss cohort study of first-degree relatives of patients with rheumatoid arthritis. BMJ Open (2021) 11(7):e048409. doi: 10.1136/bmjopen-2020-048409 PMC8280908 34261688

[74] Gupta VK Cunningham KY Hur B Bakshi U Huang H Warrington KJ. Gut microbial determinants of clinically important improvement in patients with rheumatoid arthritis. Genome Med (2021) 13(1):149. doi: 10.1186/s13073-021-00957-0 34517888 PMC8439035

[75] Wang H Ong E Kao JY Sun D He Y. Reverse microbiomics: A new reverse dysbiosis analysis strategy and its usage in prediction of autoantigens and virulent factors in dysbiotic gut microbiomes from rheumatoid arthritis patients. Front Microbiol (2021) 12:633732. doi: 10.3389/fmicb.2021.633732 33717026 PMC7947680

[76] Volkova A Ruggles KV. Predictive metagenomic analysis of autoimmune disease identifies robust autoimmunity and disease specific microbial signatures. Front Microbiol (2021) 12:621310. doi: 10.3389/fmicb.2021.621310 33746917 PMC7969817

[77] Lin SH Chang YS Lin TM Hu LF Hou TY Hsu HC. Proton pump inhibitors increase the risk of autoimmune diseases: A nationwide cohort study. Front Immunol (2021) 12:736036. doi: 10.3389/fimmu.2021.736036 34659225 PMC8514990

[78] Wang C Segal LN Hu J Zhou B Hayes RB Ahn J. Microbial risk score for capturing microbial characteristics, integrating multi-omics data, and predicting disease risk. Microbiome (2022) 10(1):121. doi: 10.1186/s40168-022-01310-2 35932029 PMC9354433

[79] Kim DS Kwon JE Lee SH Kim EK Ryu JG Jung KA. Attenuation of rheumatoid inflammation by sodium butyrate through reciprocal targeting of HDAC2 in osteoclasts and HDAC8 in T cells. Front Immunol (2018) 9:1525. doi: 10.3389/fimmu.2018.01525 30034392 PMC6043689

[80] Takahashi D Hoshina N Kabumoto Y Maeda Y Suzuki A Tanabe H. Microbiota-derived butyrate limits the autoimmune response by promoting the differentiation of follicular regulatory T cells. EBioMedicine (2020) 58:102913. doi: 10.1016/j.ebiom.2020.102913 32711255 PMC7387783

[81] Rosser EC Piper CJM Matei DE Blair PA Rendeiro AF Orford M. Microbiota-derived metabolites suppress arthritis by amplifying aryl-hydrocarbon receptor activation in regulatory b cells. Cell Metab (2020) 31(4):837–51 e10. doi: 10.1016/j.cmet.2020.03.003 32213346 PMC7156916

[82] Abendroth A Michalsen A Ludtke R Ruffer A Musial F Dobos GJ. Changes of intestinal microflora in patients with rheumatoid arthritis during fasting or a Mediterranean diet. Forsch Komplementmed. (2010) 17(6):307–13. doi: 10.1159/000322313 21196744

[83] Hager J Bang H Hagen M Frech M Trager P Sokolova MV. The role of dietary fiber in rheumatoid arthritis patients: A feasibility study. Nutrients (2019) 11(10):2392. doi: 10.3390/nu11102392 31591345 PMC6836071

[84] Diamanti AP Panebianco C Salerno G Di Rosa R Salemi S Sorgi ML. Impact of Mediterranean diet on disease activity and gut microbiota composition of rheumatoid arthritis patients. Microorganisms (2020) 8(12):14. doi: 10.3390/microorganisms8121989 PMC7764882 33327432

[85] Artacho A Isaac S Nayak R Flor-Duro A Alexander M Koo I. The pretreatment gut microbiome is associated with lack of response to methotrexate in new-onset rheumatoid arthritis. Arthritis Rheumatol (2021) 73(6):931–42. doi: 10.1002/art.41622 PMC11293279 33314800

[86] Qiao J Zhang SX Chang MJ Cheng T Zhang JQ Zhao R. Specific enterotype of gut microbiota predicted clinical effect of methotrexate in patients with rheumatoid arthritis. Rheumatol (Oxford) (2022) keac458. doi: 10.1093/rheumatology/keac458 35946529

[87] Nayak RR Alexander M Deshpande I Stapleton-Gray K Rimal B Patterson AD. Methotrexate impacts conserved pathways in diverse human gut bacteria leading to decreased host immune activation. Cell Host Microbe (2021) 29(3):362–77 e11. doi: 10.1016/j.chom.2020.12.008 33440172 PMC7954989

[88] Fan Z Yang B Paul Ross R Stanton C Zhang F Sun J. Propionate restores disturbed gut microbiota induced by methotrexate in rheumatoid arthritis: From clinic to experiments. J King Saud Univ - Sci (2021) 33(6). doi: 10.1016/j.jksus.2021.101545

[89] Rovensky J Stancikova M Svik K Uteseny J Bauerova K Jurcovicova J. Treatment of adjuvant-induced arthritis with the combination of methotrexate and probiotic bacteria escherichia coli O83 (ColinfantA (R)). Folia Microbiol (2009) 54(4):359–63. doi: 10.1007/s12223-009-0045-2 19826925

[90] Lee HJ Zhang H Orlovich DA Fawcett JP. The influence of probiotic treatment on sulfasalazine metabolism in rat. Xenobiotica (2012) 42(8):791–7. doi: 10.3109/00498254.2012.660508 22348441

[91] Picchianti-Diamanti A Panebianco C Salemi S Sorgi ML Di Rosa R Tropea A. Analysis of gut microbiota in rheumatoid arthritis patients: Disease-related dysbiosis and modifications induced by etanercept. Int J Mol Sci (2018) 19(10):2938. doi: 10.3390/ijms19102938 30261687 PMC6213034

[92] Wang B He Y Tang J Ou Q Lin J. Alteration of the gut microbiota in tumor necrosis factor-alpha antagonist-treated collagen-induced arthritis mice. Int J Rheum Dis (2020) 23(4):472–9. doi: 10.1111/1756-185X.13802 32100456

