# Supplementary material for: Relationship between gut microbiota and rheumatoid arthritis: A bibliometric analysis
Source: Front Immunol. 2023 Mar 1;14:1131933. doi: 10.3389/fimmu.2023.1131933 (PMC10015446; doi:10.3389/fimmu.2023.1131933)
Supplement: Supplementary file 2 [file Table_1.docx]

Supplementary Material 2

Relationship between gut microbiota and rheumatoid arthritis: A bibliometric analysis

Ying Dong^1^, Jianling Yao^1^, Qingyue Deng^1^, Xianxian Li^1^, Yingyu He^1^, Xueyang Ren^1^, Yuan Zheng^1^, Ruolan Song^1^, Xiangjian Zhong^1^, Jiamu Ma^1^, Dongjie Shan^1^, Fang Lv^1^, Xiuhuan Wang^1, 2^, Ruijuan Yuan^1^ * †, Gaimei She^1^ * †

†Ruijuan Yuan^1^ * † and Gaimei She^1^ *†These authors contributed equally to this work and share last authorship

^1^School of Chinese Materia Medica, Beijing University of Chinese Medicine, Beijing 102488, China.

^2^Peking University HuiLongGuan Clinical Medical School, Beijing Huilongguan Hospital, Beijing, 100096, P. R. China

*** Correspondence:**Ruijuan Yuan
rjyuance@126.com

Gaimei She
shegaimei@126.com

Table S1 Gut microbes may be related to RA

| Microbes | | | | | | | Research object | Performance in RA group | Reference |
| --- | --- | --- | --- | --- | --- | --- | --- | --- | --- |
| kingdom | phylum | class | order | family | genus | species |  |  |  |
| Bacteria | Actinobacteria |  |  |  |  |  | feces | increase | (1, 2) |
|  |  |  |  |  |  |  | feces | decrease | (3, 4) |
|  |  |  | Actinomycetales |  |  |  | feces | increase | (1) |
|  |  |  |  | Actinomycetaceae |  |  | feces | increase | (1) |
|  |  |  |  |  | Actinomyces |  | feces | increase | (1, 5) |
|  |  |  |  |  |  | A. naeslundii | synovial tissue | detected | (6) |
|  |  |  |  | Nocardiaceae |  |  |  |  |  |
|  |  |  |  |  | Rhodococcus |  | feces | decrease | (7) |
|  |  |  |  |  |  | R. fascians | synovial tissue | detected | (6) |
|  |  |  |  | Corynebacteriaceae |  |  |  |  |  |
|  |  |  |  |  | Corynebacterium |  | feces | increase | (8) |
|  |  |  |  |  |  | C. aurimucosum | synovial tissue | detected | (6) |
|  |  |  |  | Micrococcaceae |  |  | feces | increase | (9) |
|  |  |  |  |  | Rothia |  | feces | increase | (5, 9) |
|  |  |  | Bifidobacteriales |  |  |  |  |  |  |
|  |  |  |  | Bifidobacteriaceae |  |  | feces | decrease | (10) |
|  |  |  |  |  |  |  | feces | increase | (11) |
|  |  |  |  |  | Bifidobacterium |  | feces | decrease | (12, 13) |
|  |  |  |  |  |  |  | feces | increase | (11) |
|  |  |  |  |  |  | B. bifidum | feces | decrease | (14) |
|  |  |  |  |  |  | B. dentium | feces | increase | (14) |
|  |  |  |  |  |  | B. adolescentis | feces | decrease | (15) |
|  |  |  |  |  | Gardnerella |  | feces | increase | (16) |
|  |  |  |  |  |  | G. vaginalis | feces | increase | (16) |
|  |  |  | Coriobacteriales |  |  |  | feces | increase | (1) |
|  |  |  |  |  |  |  | feces | decrease | (3, 15) |
|  |  |  |  | Coriobacteriaceae |  |  | feces | decrease | (3, 15) |
|  |  |  |  |  | Gordonibacter |  |  |  |  |
|  |  |  |  |  |  | G. pamelaeae | feces | increase | (14) |
|  |  |  |  |  | Eggerthella |  | feces | increase | (1, 5, 9) |
|  |  |  |  |  |  | E. lenta | feces | increase | (14) |
|  |  |  |  |  | Collinsella |  | feces | decrease | (3, 4) |
|  |  |  |  |  |  |  | feces | increase | (8) |
|  |  |  |  |  |  | C. aerofaciens | feces | increase | (17) |
|  |  |  |  |  | Adlercreutzia |  | feces | increase | (8) |
|  | Firmicutes |  |  |  |  |  | feces | decrease | (15, 18) |
|  |  | Bacilli |  |  |  |  | feces | increase | (1, 9) |
|  |  |  | Lactobacillales |  |  |  | feces | increase | (1, 9) |
|  |  |  |  | Lactobacillaceae |  |  | feces | increase | (18) |
|  |  |  |  |  | Lactobacillus |  | feces | increase | (13, 18, 19) |
|  |  |  |  |  |  |  | feces | decrease | (7, 12) |
|  |  |  |  |  |  | L. salivarius | feces | increase | (14, 19) |
|  |  |  |  |  |  | L. iners | feces | increase | (19) |
|  |  |  |  |  |  | L. ruminis | feces | increase | (15, 19) |
|  |  |  |  |  |  | L. mucosae | feces | detected | (19) |
|  |  |  |  |  |  | L. sp. | feces | increase | (14) |
|  |  |  |  |  | Roseburia |  | feces | decrease | (9, 13) |
|  |  |  |  |  |  |  | feces | increase | (8) |
|  |  |  |  | Leuconostocaceae |  |  | feces | increase | (9) |
|  |  |  |  |  | Weissella |  | feces | increase | (9) |
|  |  |  |  | Streptococcaceae |  |  | feces | increase | (1, 9, 20) |
|  |  |  |  |  | Streptococcus |  | feces | increase | (1, 5, 9, 13, 20) |
|  |  |  |  |  |  | S. thermophilus | synovial tissue | detected | (6) |
|  |  |  |  | Enterococcaceae |  |  |  |  |  |
|  |  |  |  |  | Enterococcus |  | feces | decrease | (7) |
|  |  |  | Bacillales |  |  |  |  |  |  |
|  |  |  |  |  | Gemella |  | feces | increase | (5) |
|  |  |  |  | Staphylococcaceae |  |  |  |  |  |
|  |  |  |  |  | Staphylococcus |  | feces | decrease | (7) |
|  |  |  |  | Thermoactinomycetaceae |  |  | feces | decrease | (9) |
|  |  |  |  |  | Kroppenstedtia |  | feces | decrease | (9) |
|  |  | Negativicutes |  |  |  |  | feces | decrease | (18) |
|  |  |  | Selenomonadales |  |  |  | feces | increase | (18, 21) |
|  |  |  |  |  |  |  | feces | decrease | (15) |
|  |  |  |  | Selenomonadaceae |  |  | feces | decrease | (11, 15) |
|  |  |  |  | Veillonellaceae |  |  | feces | decrease | (18) |
|  |  |  |  |  | Veillonella |  | feces | increase | (22) |
|  |  |  |  |  |  |  | feces | decrease | (14) |
|  |  |  |  |  |  | V. seminalis | feces | decrease | (15) |
|  |  |  |  |  | Megamonas |  | feces | decrease | (11, 13, 15, 18, 21) |
|  |  |  |  |  |  |  | feces | increase | (8, 18) |
|  |  |  |  |  |  | M. hypermegale | feces | decrease | (14) |
|  |  |  |  |  | Dialister |  | feces | decrease | (18) |
|  |  |  |  |  |  |  | feces | increase | (8, 11) |
|  |  |  |  |  |  | D. invisus | feces | decrease | (21) |
|  |  |  |  | Acidaminococcaceae |  |  |  |  |  |
|  |  |  |  |  | Phascolarctobacterium |  | feces | decrease | (22) |
|  |  |  |  |  |  |  | feces | increase | (23) |
|  |  | Erysipelotrichia |  |  |  |  | feces | decrease | (3) |
|  |  |  | Erysipelotrichales |  |  |  | feces | decrease | (3) |
|  |  |  |  | Erysipelotrichaceae |  |  | feces | decrease | (3) |
|  |  |  |  |  | Catenibacterium |  | feces | increase | (21, 22) |
|  |  |  |  |  | Turicibacter |  | feces | increase | (1) |
|  |  | Clostridia |  |  |  |  | feces | decrease | (18) |
|  |  |  | Clostridiales |  |  |  | feces | increase | (23) |
|  |  |  |  |  |  |  | feces | decrease | (18) |
|  |  |  |  |  | Blautia |  | feces | decrease | (18) |
|  |  |  |  |  |  |  | feces | increase | (11, 20) |
|  |  |  |  |  |  | B. gnavus | feces | increase | (24) |
|  |  |  |  |  | Gallicola |  | feces | decrease | (9) |
|  |  |  |  | Clostridiaceae |  |  | feces | decrease | (25) |
|  |  |  |  |  | Clostridium |  | feces | decrease | (4, 25) |
|  |  |  |  |  |  |  | feces | increase | (23) |
|  |  |  |  |  |  | C. asparagiforme | feces | increase | (14) |
|  |  |  |  |  |  | C.leptum | feces | decrease | (21, 26) |
|  |  |  |  |  | Butyricicoccus |  | feces | decrease | (5) |
|  |  |  |  | Lachnospiraceae |  |  | feces | increase | (14) |
|  |  |  |  |  |  |  | feces | decrease | (18) |
|  |  |  |  |  | Lachnospira |  | feces | decrease | (5, 11) |
|  |  |  |  |  | Roseburia |  | feces | decrease | (5, 18) |
|  |  |  |  |  |  | R. faecis | feces | decrease | (15) |
|  |  |  |  |  | Dorea |  |  |  |  |
|  |  |  |  |  |  | D. formicigenerans | feces | decrease | (17) |
|  |  |  |  |  | Coprococcus |  | feces | decrease | (11, 18) |
|  |  |  |  |  | Anaerostipes |  | feces | decrease | (18) |
|  |  |  |  | Ruminococcaceae |  |  | feces | increase | (11, 20) |
|  |  |  |  |  |  |  | feces | decrease | (4) |
|  |  |  |  |  | Ruminococcus |  | feces | increase | (8) |
|  |  |  |  |  |  | R. lactaris | feces | increase | (14) |
|  |  |  |  |  | Faecalibacterium |  | feces | increase | (1, 5, 9, 13, 20) |
|  |  |  |  |  |  |  | feces | decrease | (5) |
|  |  |  |  |  | Sporobacter |  | feces | decrease | (5) |
|  |  |  |  |  | Subdoligranulum |  | feces | decrease | (5) |
|  |  |  |  |  | Anaerotruncus |  |  |  |  |
|  |  |  |  |  |  | A. colihominis | feces | decrease | (21) |
|  |  |  |  |  | Acetivibrio |  | feces | decrease | (18) |
|  |  |  |  |  | Acetanaerobacterium |  |  |  |  |
|  |  |  |  |  |  | A. elongatum | feces | decrease | (24) |
|  |  |  |  | Eubacteriaceae |  |  | feces | increase | (1) |
|  |  |  |  |  | Eubacterium |  | feces | increase | (1, 8) |
|  |  |  |  |  | Anaerofustis |  | feces | decrease | (5) |
|  |  |  |  | Christensenellaceae |  |  |  |  |  |
|  |  |  |  |  | Christensenella |  |  |  |  |
|  |  |  |  |  |  | C. minuta | feces | decrease | (21) |
|  |  |  |  |  |  | C. massiliensis | feces | decrease | (24) |
|  |  |  |  | Peptostreptococcaceae |  |  | feces | decrease | (21) |
|  |  |  |  |  |  |  | feces | increase | (11) |
|  |  |  |  |  | Fusobacterium |  | feces | decrease | 10.1155/2021/6665563 |
|  |  |  |  | Oscillospiraceae |  |  | feces | increase | (11) |
|  |  |  |  | Gracilibacteraceae |  |  |  |  |  |
|  |  |  |  |  | Gracilibacter |  |  |  |  |
|  |  |  |  |  |  | G. thermotolerans | feces | decrease | (24) |
|  | Proteobacteria |  |  |  |  |  | feces | increase | (10, 15) |
|  |  | Alphaproteobacteria |  |  |  |  | feces | increase | (18) |
|  |  |  | Caulobacterales |  |  |  |  |  |  |
|  |  |  |  | Caulobacteraceae |  |  |  |  |  |
|  |  |  |  |  | Caulobacter |  |  |  |  |
|  |  |  |  |  |  | Caulobacter sp. | synovial tissue | detected | (6) |
|  |  |  | Rhizobiales |  |  |  |  |  |  |
|  |  |  |  | Hyphomicrobiaceae |  |  |  |  |  |
|  |  |  |  |  | Gemmiger |  | feces | decrease | (5, 18) |
|  |  | Gammaproteobacteria |  |  |  |  | feces | increase | (15, 18) |
|  |  |  | Enterobacteriales |  |  |  |  |  |  |
|  |  |  |  | Enterobacteriaceae |  |  | feces | increase | (10, 15, 18) |
|  |  |  |  |  | Enterobacter |  | feces | decrease | (7) |
|  |  |  |  |  | Serratia |  |  |  |  |
|  |  |  |  |  |  | S. proteamaculans | synovial tissue | detected | (6) |
|  |  |  |  |  | Shigella |  |  |  |  |
|  |  |  |  |  |  | S. sp. | synovial tissue | detected | (6) |
|  |  |  |  |  | Escherichia |  | feces | increase | (15) |
|  |  |  |  |  |  | Escherichia coli | synovial tissue | detected | (6) |
|  |  |  |  |  |  |  | feces | increase | (12) |
|  |  |  |  |  | Klebsiella |  | feces | increase | (10, 15) |
|  |  |  |  |  |  |  | feces | decrease | (7, 23) |
|  |  |  |  |  |  | K. pneumoniae | feces | decrease | (14) |
|  |  |  |  |  |  |  | feces | increase | (15) |
|  |  |  |  |  | Erwinia |  | feces | increase | (1) |
|  |  |  |  |  | Citrobacter |  | feces | decrease | (7) |
|  |  |  |  |  |  |  | feces | increase | (18) |
|  |  |  | Pseudomonadales |  |  |  | feces | increase | (9) |
|  |  |  |  | Pseudomonadaceae |  |  | feces | increase | (9) |
|  |  |  |  |  | Pseudomonas |  | feces | increase | (9) |
|  |  |  |  |  |  | P. sp. | synovial tissue | detected | (6) |
|  |  |  |  | Moraxellaceae |  |  |  |  |  |
|  |  |  |  |  | Acinetobacter |  |  |  |  |
|  |  |  |  |  |  | A. sp. | synovial tissue | detected | (6) |
|  |  |  | Pasteurellales |  |  |  |  |  |  |
|  |  |  |  | Pasteurellaceae |  |  |  |  |  |
|  |  |  |  |  | Haemophilus |  | feces | decrease | (14) |
|  |  |  | Aeromonadales |  |  |  |  |  |  |
|  |  |  |  | Succinivibrionaceae |  |  | feces | increase | (10) |
|  |  |  |  |  |  |  |  |  |  |
|  |  | Betaproteobacteria |  |  |  |  | feces | decrease | (18) |
|  |  |  | Burkholderiales |  |  |  | feces | decrease | (18) |
|  |  |  |  | Alcaligenaceae |  |  |  |  |  |
|  |  |  |  |  | Alcaligenes |  |  |  |  |
|  |  |  |  |  |  | A. faecalis | synovial tissue | detected | (6) |
|  |  |  |  | Sutterellaceae |  |  | feces | decrease | (11, 18) |
|  |  |  |  |  | Sutterella |  | feces | decrease | (11) |
|  |  |  |  |  |  | S. wadsworthensis | feces | decrease | (14) |
|  |  |  |  |  | Parasutterella |  | feces | decrease | (18) |
|  |  | Deltaproteobacteria |  |  |  |  |  |  |  |
|  |  |  | Desulfovibrionales |  |  |  |  |  |  |
|  |  |  |  | Desulfovibrionaceae |  |  | feces | increase | (10) |
|  |  |  |  |  | Desulfovibrio |  | feces | decrease | (7) |
|  |  |  |  |  | Bilophila |  | feces | increase | (8) |
|  |  | Epsilonproteobacteria |  |  |  |  |  |  |  |
|  |  |  | Campylobacterales |  |  |  |  |  |  |
|  |  |  |  | Helicobacteraceae |  |  |  |  |  |
|  |  |  |  |  | Helicobacter |  | feces | decrease | (7) |
|  |  |  |  |  |  |  |  |  |  |
|  |  |  |  |  | Prevotella |  | feces | increase | (22, 26) |
|  |  |  |  |  | Odoribacter |  | feces | decrease | (7, 11) |
|  |  |  |  |  | Butyricimonas |  | feces | decrease | (11) |
|  |  |  |  |  | Bacteroides |  | feces | decrease | (11, 13, 22) |
|  |  |  |  |  | Sphingobacterium |  |  |  |  |
|  |  |  |  |  | Rikenella |  | feces | decrease | (7) |
|  |  |  |  |  | Porphyromonas |  |  |  |  |
|  |  |  |  |  | Flavobacterium |  | feces | increase | (15) |
|  |  |  |  |  | Alistipes |  | feces | decrease | (21) |
|  | Bacteroidetes |  |  |  |  |  | feces | increase | (3) |
|  |  |  |  |  |  |  | feces | decrease | (21) |
|  |  | Bacteroidia |  |  |  |  | feces | increase | (3) |
|  |  |  |  |  |  |  | feces | decrease | (21) |
|  |  |  | Bacteroidales |  |  |  | feces | increase | (3) |
|  |  |  |  |  |  |  | feces | decrease | (20, 21) |
|  |  |  |  | Prevotellaceae |  |  | feces | increase | (22) |
|  |  |  |  |  |  |  | feces | decrease | (10, 20) |
|  |  |  |  |  |  |  | feces | decrease | (11, 18) |
|  |  |  |  |  |  | P. copri | feces | increase | (22, 27) |
|  |  |  |  |  |  | P. denticola | feces | increase | (16) |
|  |  |  |  |  |  | P. marshii | feces | increase | (16) |
|  |  |  |  |  |  | P. disiens | feces | increase | (16) |
|  |  |  |  |  |  | P. corporis | feces | increase | (16) |
|  |  |  |  |  |  | P. amnii | feces | increase | (16) |
|  |  |  |  | Bacteroidaceae |  |  | feces | decrease | (11, 22) |
|  |  |  |  |  |  |  | feces | increase | (7, 8, 26) |
|  |  |  |  |  |  | B. faecichinchillae | feces | decrease | (21) |
|  |  |  |  |  |  | B. acidifaciens | feces | decrease | (21) |
|  |  |  |  |  |  | B. sartorii | feces | increase | (16) |
|  |  |  |  | Rikenellaceae |  |  | feces | decrease | (11, 21) |
|  |  |  |  | Porphyromonadaceae |  |  |  |  |  |
|  |  |  |  |  |  | P. somerae | feces | increase | (16) |
|  |  | Sphingobacteriia |  |  |  |  |  |  |  |
|  |  |  | Sphingobacteriales |  |  |  |  |  |  |
|  |  |  |  | Sphingobacteriaceae |  |  |  |  |  |
|  |  |  |  |  | Sphingobacterium |  |  |  |  |
|  |  |  |  |  |  | S. mizutaii | synovial tissue | detected | (6) |
|  |  | Flavobacteriia |  |  |  |  |  |  |  |
|  |  |  | Flavobacteriales |  |  |  |  |  |  |
|  |  |  |  | Flavobacteriaceae |  |  |  |  |  |
|  |  |  |  |  |  |  |  |  |  |
|  | Verrucomicrobia |  |  |  |  |  |  |  |  |
|  |  | Verrucomicrobiae |  |  |  |  | feces | decrease | (20) |
|  |  |  | Verrucomicrobiales |  |  |  |  |  |  |
|  |  |  |  | Akkermansiaceae |  |  | feces | decrease | (20) |
|  |  |  |  |  | Akkermansia |  | feces | decrease | (7, 20) |
|  |  |  |  |  |  |  | feces | increase | (23) |
|  | Tenericutes |  |  |  |  |  |  |  |  |
|  |  |  | Mycoplasmatales |  |  |  | feces | decrease | (9) |
|  |  |  |  | Mycoplasmataceae |  |  | feces | decrease | (9) |
|  |  |  |  |  | Mycoplasma |  | feces | decrease | (9) |
|  | Synergistetes |  |  |  |  |  |  |  |  |
|  |  | Synergistia |  |  |  |  | feces | decrease | (18) |
|  |  |  | Synergistales |  |  |  | feces | decrease | (18) |
|  |  |  |  | Synergistaceae |  |  | feces | decrease | (18) |
|  | Fusobacteria |  |  |  |  |  | feces | decrease | (18) |
|  |  | Fusobacteriia |  |  |  |  | feces | decrease | (18) |
|  |  |  | Fusobacteriales |  |  |  | feces | decrease | (18) |
|  |  |  |  | Fusobacteriaceae |  |  | feces | decrease | (18) |
| Fungi | Ascomycota |  |  |  |  |  |  |  |  |
|  |  | Dothideomycetes |  |  |  |  |  |  |  |
|  |  |  | Pleosporales |  |  |  | feces | decrease | (28) |
|  |  |  |  | Pleosporaceae |  |  |  |  |  |
|  |  |  |  |  | Alternaria |  | feces | decrease | (28) |
|  |  |  | Capnodiales |  |  |  |  |  |  |
|  |  |  |  |  | Scolecostigmina |  | feces | increase | (28) |
|  |  |  |  |  |  |  |  |  |  |
|  |  |  |  |  |  |  |  |  |  |
|  |  | Saccharomycetes |  |  |  |  |  |  |  |
|  |  |  | Saccharomycetales |  |  |  |  |  |  |
|  |  |  |  |  | Aciculoconidium |  | feces | decrease | (28) |
|  |  |  |  | Dipodascaceae |  |  | feces | decrease | (28) |
|  |  |  |  |  | Dipodascus |  | feces | decrease | (28) |
|  |  | Leotiomycetes |  |  |  |  |  |  |  |
|  |  |  | Erysiphales |  |  |  |  |  |  |
|  |  |  |  | Erysiphaceae |  |  |  |  |  |
|  |  |  |  |  | Podosphaera |  | feces | decrease | (28) |
|  |  | Sordariomycetes |  |  |  |  |  |  |  |
|  |  |  | Hypocreales |  |  |  |  |  |  |
|  |  |  |  |  | Sarocladium |  | feces | increase | (28) |
|  |  |  |  | Ophiocordycipitaceae |  |  |  |  |  |
|  |  |  |  |  | Tolypocladium |  | feces | increase | (28) |
|  | Basidiomycota |  |  |  |  |  |  |  |  |
|  |  | Agaricomycetes |  |  |  |  |  |  |  |
|  |  |  | Polyporales |  |  |  |  |  |  |
|  |  |  |  | Ganodermataceae |  |  |  |  |  |
|  |  |  |  |  | Ganoderma |  | feces | decrease | (28) |
|  |  |  |  | Phanerochaetaceae |  |  |  |  |  |
|  |  |  |  |  | Phanerochaete |  | feces | increase | (28) |
|  |  |  | Auriculariales |  |  |  | feces | decrease | (28) |
|  |  |  |  | Auriculariaceae |  |  | feces | decrease | (28) |
|  |  |  |  |  | Auricularia |  | feces | decrease | (28) |
|  |  |  | Agaricales |  |  |  |  |  |  |
|  |  |  |  | Strophariaceae |  |  | feces | decrease | (28) |
|  |  |  |  |  | Pholiota |  | feces | decrease | (28) |
|  |  |  |  | Pleurotaceae |  |  | feces | decrease | (28) |
|  |  |  |  |  | Pleurotus |  | feces | decrease | (28) |
|  |  | Wallemiomycetes |  |  |  |  | feces | increase | (28) |
|  |  |  | Wallemiales |  |  |  | feces | increase | (28) |
|  |  |  |  | Wallemiaceae |  |  |  |  |  |
|  |  |  |  |  | Wallemia |  | feces | increase | (28) |

# REFERENCES

1. Chen J, Wright K, Davis JM, Jeraldo P, Marietta EV, Murray J, et al. An expansion of rare lineage intestinal microbes characterizes rheumatoid arthritis. Genome Med. 2016;8(1):43.

2. Ruiz-Limon P, Mena-Vazquez N, Moreno-Indias I, Manrique-Arija S, Lisbona-Montanez JM, Cano-Garcia L, et al. Collinsella is associated with cumulative inflammatory burden in an established rheumatoid arthritis cohort. Biomed Pharmacother. 2022;153:113518.

3. Jeong Y, Kim JW, You HJ, Park SJ, Lee J, Ju JH, et al. Gut Microbial Composition and Function Are Altered in Patients with Early Rheumatoid Arthritis. J Clin Med. 2019;8(5).

4. Jeong Y, Jhun J, Lee SY, Na HS, Choi J, Cho KH, et al. Therapeutic Potential of a Novel Bifidobacterium Identified Through Microbiome Profiling of RA Patients With Different RF Levels. Front Immunol. 2021;12:736196.

5. Forbes JD, Chen CY, Knox NC, Marrie RA, El-Gabalawy H, de Kievit T, et al. A comparative study of the gut microbiota in immune-mediated inflammatory diseases-does a common dysbiosis exist? Microbiome. 2018;6(1):221.

6. Siala M, Jaulhac B, Gdoura R, Sibilia J, Fourati H, Younes M, et al. Analysis of bacterial DNA in synovial tissue of Tunisian patients with reactive and undifferentiated arthritis by broad-range PCR, cloning and sequencing. Arthritis Res Ther. 2008;10(2):R40.

7. Sun Y, Chen Q, Lin P, Xu R, He D, Ji W, et al. Characteristics of Gut Microbiota in Patients With Rheumatoid Arthritis in Shanghai, China. Front Cell Infect Microbiol. 2019;9:369.

8. El Menofy NG, Ramadan M, Abdelbary ER, Ibrahim HG, Azzam AI, Ghit MM, et al. Bacterial Compositional Shifts of Gut Microbiomes in Patients with Rheumatoid Arthritis in Association with Disease Activity. Microorganisms. 2022;10(9).

9. Liu Z, Wu Y, Luo Y, Wei S, Lu C, Zhou Y, et al. Self-Balance of Intestinal Flora in Spouses of Patients With Rheumatoid Arthritis. Front Med (Lausanne). 2020;7:538.

10. Breban M, Tap J, Leboime A, Said-Nahal R, Langella P, Chiocchia G, et al. Faecal microbiota study reveals specific dysbiosis in spondyloarthritis. Ann Rheum Dis. 2017;76(9):1614-22.

11. Wang Q, Zhang SX, Chang MJ, Qiao J, Wang CH, Li XF, et al. Characteristics of the Gut Microbiome and Its Relationship With Peripheral CD4(+) T Cell Subpopulations and Cytokines in Rheumatoid Arthritis. Front Microbiol. 2022;13:799602.

12. Yong W, Hongbin L, Jing W, Jing Z, Ning T, Lijie B. Associations of Changes in Serum Inflammatory Factors, MMP-3, 25(OH)D and Intestinal Flora with Osteoporosis and Disease Activity in Rheumatoid Arthritis Patients. Clin Lab. 2020;66(12).

13. Zhu J, Wang T, Lin Y, Xiong M, Chen J, Jian C, et al. The change of plasma metabolic profile and gut microbiome dysbiosis in patients with rheumatoid arthritis. Front Microbiol. 2022;13:931431.

14. Zhang X, Zhang D, Jia H, Feng Q, Wang D, Liang D, et al. The oral and gut microbiomes are perturbed in rheumatoid arthritis and partly normalized after treatment. Nat Med. 2015;21(8):895-905.

15. Yu D, Du J, Pu X, Zheng L, Chen S, Wang N, et al. The Gut Microbiome and Metabolites Are Altered and Interrelated in Patients With Rheumatoid Arthritis. Front Cell Infect Microbiol. 2021;11:763507.

16. Kishikawa T, Maeda Y, Nii T, Motooka D, Matsumoto Y, Matsushita M, et al. Metagenome-wide association study of gut microbiome revealed novel aetiology of rheumatoid arthritis in the Japanese population. Ann Rheum Dis. 2020;79(1):103-11.

17. Mena-Vazquez N, Ruiz-Limon P, Moreno-Indias I, Manrique-Arija S, Tinahones FJ, Fernandez-Nebro A. Expansion of Rare and Harmful Lineages is Associated with Established Rheumatoid Arthritis. J Clin Med. 2020;9(4).

18. Li Y, Zhang SX, Yin XF, Zhang MX, Qiao J, Xin XH, et al. The Gut Microbiota and Its Relevance to Peripheral Lymphocyte Subpopulations and Cytokines in Patients with Rheumatoid Arthritis. J Immunol Res. 2021;2021:6665563.

19. Liu X, Zou Q, Zeng B, Fang Y, Wei H. Analysis of fecal Lactobacillus community structure in patients with early rheumatoid arthritis. Curr Microbiol. 2013;67(2):170-6.

20. Chen YM, Ma CY, Liu LX, He JQ, Zhu CX, Zheng FP, et al. Analysis of gut microbiota and metabolites in patients with rheumatoid arthritis and identification of potential biomarkers. Aging-US. 2021;13(20):23689-701.

21. Lee JY, Mannaa M, Kim Y, Kim J, Kim GT, Seo YS. Comparative Analysis of Fecal Microbiota Composition Between Rheumatoid Arthritis and Osteoarthritis Patients. Genes (Basel). 2019;10(10).

22. Scher JU, Sczesnak A, Longman RS, Segata N, Ubeda C, Bielski C, et al. Expansion of intestinal Prevotella copri correlates with enhanced susceptibility to arthritis. Elife. 2013;2:e01202.

23. Chiang HI, Li JR, Liu CC, Liu PY, Chen HH, Chen YM, et al. An Association of Gut Microbiota with Different Phenotypes in Chinese Patients with Rheumatoid Arthritis. J Clin Med. 2019;8(11).

24. Marazzato M, Iannuccelli C, Guzzo MP, Nencioni L, Lucchino B, Radocchia G, et al. Gut Microbiota Structure and Metabolites, Before and After Treatment in Early Rheumatoid Arthritis Patients: A Pilot Study. Front Med (Lausanne). 2022;9:921675.

25. Muniz Pedrogo DA, Chen J, Hillmann B, Jeraldo P, Al-Ghalith G, Taneja V, et al. An Increased Abundance of Clostridiaceae Characterizes Arthritis in Inflammatory Bowel Disease and Rheumatoid Arthritis: A Cross-sectional Study. Inflamm Bowel Dis. 2019;25(5):902-13.

26. Rodrigues GSP, Cayres LCF, Goncalves FP, Takaoka NNC, Lengert AH, Tansini A, et al. Detection of Increased Relative Expression Units of Bacteroides and Prevotella, and Decreased Clostridium leptum in Stool Samples from Brazilian Rheumatoid Arthritis Patients: A Pilot Study. Microorganisms. 2019;7(10).

27. Maeda Y, Kurakawa T, Umemoto E, Motooka D, Ito Y, Gotoh K, et al. Dysbiosis Contributes to Arthritis Development via Activation of Autoreactive T Cells in the Intestine. Arthritis Rheumatol. 2016;68(11):2646-61.

28. Sun X, Wang Y, Li X, Wang M, Dong J, Tang W, et al. Alterations of gut fungal microbiota in patients with rheumatoid arthritis. PeerJ. 2022;10:e13037.
